# Population Structure, Genetic Diversity and Molecular Marker-Trait Association Analysis for High Temperature Stress Tolerance in Rice

**DOI:** 10.1371/journal.pone.0160027

**Published:** 2016-08-05

**Authors:** Sharat Kumar Pradhan, Saumya Ranjan Barik, Ambika Sahoo, Sudipti Mohapatra, Deepak Kumar Nayak, Anumalla Mahender, Jitandriya Meher, Annamalai Anandan, Elssa Pandit

**Affiliations:** Crop Improvement Division, ICAR-National Rice Research Institute, Cuttack, India; National Institute of Plant Genome Research, INDIA

## Abstract

Rice exhibits enormous genetic diversity, population structure and molecular marker-traits associated with abiotic stress tolerance to high temperature stress. A set of breeding lines and landraces representing 240 germplasm lines were studied. Based on spikelet fertility percent under high temperature, tolerant genotypes were broadly classified into four classes. Genetic diversity indicated a moderate level of genetic base of the population for the trait studied. Wright’s F statistic estimates showed a deviation of Hardy-Weinberg expectation in the population. The analysis of molecular variance revealed 25 percent variation between population, 61 percent among individuals and 14 percent within individuals in the set. The STRUCTURE analysis categorized the entire population into three sub-populations and suggested that most of the landraces in each sub-population had a common primary ancestor with few admix individuals. The composition of materials in the panel showed the presence of many QTLs representing the entire genome for the expression of tolerance. The strongly associated marker RM547 tagged with spikelet fertility under stress and the markers like RM228, RM205, RM247, RM242, INDEL3 and RM314 indirectly controlling the high temperature stress tolerance were detected through both mixed linear model and general linear model TASSEL analysis. These markers can be deployed as a resource for marker-assisted breeding program of high temperature stress tolerance.

## Introduction

Rice is the staple food of nearly 3.5 billion consumers with a total paddy production of 715 million tons in the world [1]. The rapid phase of urbanization and industrialization erodes the environment by increasing the level of greenhouse gas leading to global warming. Thus, the high temperature stress has become a major disastrous factor that impedes dry season rice productivity in the countries of sub-tropical region. The tropical and subtropical countries, such as India, Pakistan, Bangladesh, China, Thailand, Sudan, and some other African countries are obviously encountering yield loss due to high temperature stress [2,3]. Earlier report indicated that 3 million ha of rice was damaged during 2003 accruing a loss of 5.18 million tons of rice in China due to the prevalence of continuous high temperature (>38°C) for more than 20 days [4–6]. Heat stress can be considered as a serious threat to sustaining rice production in the most productive regions of tropical and sub-tropical Asia. The global average temperature has increased by 0.6°C over the past 100 years and is projected to increase at a rapid rate in future [7]. The average increase is expected to be 0.5–2.8°C by the end of the 21^st^ century [8,9]. Studies on the effect of high temperature stress on grain yield revealed a decrease of 10% rice grain yield by a rise of 1°C temperature [10]. Among different growth stages of rice, the flowering stage is the most sensitive stage to high temperature [11,12]. The temperature beyond 35°C during flowering stage adversely affects high pollen and spikelet fertility.However, high temperature hasmore deleterious effect on just before or during anthesis [12]. Further, it affects anther dehiscence, pollination, and pollengermination, thereby leading to spikeletsterility and yield loss [13]. Exposure of rice spikelet during anthesis even for less than an hourat 33.7°C may result in spikelet sterility[14].

The heat tolerant genotypes dehisce anther seamlessly under high temperature than the susceptible ones [12,15,16,17]. Therefore, the estimation ofspikelet fertility (SF) can be used as a screening tool for tolerance to high temperature stress during the flowering stage [18]. Genotypes endowed to withstand high temperature during anthesis result in high spikelet fertility which can be of great asset to develop high temperature stress tolerant lines by associating stress phenotypes with the chromosomal regions of the tolerant genotypes. Genotypic variation in high temperature stress tolerance during flowering stage is reported in *indica* and *japonica* sub-species [13,14,17,18,19,20]. Nagina 22 (N22), a known drought tolerant genotype of India, is highly tolerant to high temperature stress [21]. The genetic variation in high temperature stress tolerance is the pre-requisite for high temperature tolerant breeding program. Molecular markers, especially SSR or Simple Sequence Length Polymorphism (SSLP) markers have been used for estimating rice genetic diversity [22–30]. For selection of suitable diverse parental lines possessing temperature tolerance, the genetic diversity pattern should be estimated by using the trait linked SSR markers that would confirm the suitability of the material to be used in the breeding program. The genetic diversity and structure of the population are imperative for association mapping and molecular breeding program for the trait of concern. For a perfect association analysis, the population should not show unauthentic association or unequal relatedness within the population [14]. The population structure (*Q*) with relative kinship (*K*) matrices is used to correct the linkage disequilibrium (LD) due to population structure and familial relatedness for mixed-model association mapping [31]. Thus, marker-based kinship estimate is considered appropriate for association mapping approaches that have shown to perform better than other alternatives.

The association between DNA haplotypes and a particular phenotype may allow the assessment of genetic potential of the donors and breeding lines that are to be used in the breeding program. Several reports on QTL mapping for heat tolerance at flowering stages have been conducted using bi-parental mapping populations [21,32–36]. This type of mapping is much costlier and only a few alleles can be identified over a long research timescale with low resolution [37–40]. These limitations are now reduced with the use of association mapping or linkage disequilibrium (LD) mapping. The main aim of LD mapping is to estimate the correlations between genotypes and phenotypes in a sample of individuals of a disequilibrium population. The association mapping for various traits is reported in many crops, like maize [41], wheat [42,43], barley [44], oilseed rape [45], *Brassica rapa* [46], soybean [47], *G*. *hirsutum* [48] and rice [49–52].Therefore, the objective of the present study is to estimate the diversity, population structure and to identify associated markers with high temperature stress tolerance traits in rice.

## Materials and Methods

### Plant materials and field screening of genotypes for high temperature stress tolerance

A total of 240 germplasm accessions, short listed on the basis of spikelet fertility, shot duration and photo-insensitivity traits during previous years were utilized for marker-trait association study(S1 Table). Thesematerials were mostly indigenous with some exotic landraces and improved cultivars obtained from National Rice Research Institute (NRRI) gene bank. Four staggered sowings at an interval of 7 days was carried out starting in mid-January, 2014 enable coincide flowering with concomitant high temperature stress during April-May. Twenty-five days old seedlings were transplanted in a staggered manner with 7 days interval following an augmented block design comprising of four check varieties (Dular, Chandan, N22 and Satyakrishna) with three rows of 4 m length each at a spacing of 20 x 15cm between hills. During flowering period, observations on daily average maximum and minimum temperature were collected from the meteorology observatory of the institute. Spikelet fertility was recorded for all the staggered plantings to infer effect of high temperature stress on magnitude of tolerance.

### Screening for high temperature stress tolerance under control and normal condition

The seeds of 60 genotypes including N22 (tolerant check) and Satyakrishna (susceptible check) were sown in plastic trays containing soil and were grown in a net house under normal condition. Twenty-one days old seedlings were uprooted and transplanted into uniform plastic pots filled with field soil and replicated thrice. One set of materials was exposed to high temperature stress in the growth chamber whereas the other set was maintained in the green house chamber to compare their relative performance under two treatment conditions. The first three heading panicles were marked and the potted plants were moved to growth chamber for high temperature treatment. The temperature in the growth chamber was set to simulate the daily ambient temperature (Table 1). The pots were brought back to the green house when all the spikelets on the three marked panicles completed flowering. At physiological maturity stage, the number of filled spikelets and chaffy spikelets were recorded. The mean spikelet fertility (percentage of filled spikelet) of the marked panicles was used as an index to evaluate the heat tolerance of the genotypes. Eleven agro-morphological traits *viz*., days to 50% flowering, plant height (cm), flag leaf length (cm), flag leaf breadth (cm), panicles/plant, panicle length (cm), spikelet fertility and sterility (%) under normal and stress condition, panicle exsertion under normal and stress condition were recorded. IRRI Standard Evaluation System to score panicle exsertion was followed. Score of 1 for well exserted; 3-moderately well exserted; 5-just exserted; 7-partially exserted and 9-enclosed were used for panicle exsertion trait. Principal Component Analysis (PCA) was performed to estimate Euclidean distance between two genotypes in the multivariate space and identification of genotypes to desired environment [53, 54]. The analyses were performed using the Windostat 7.5 software (Indostat Services, Hyderabad, India.).

**Table 1:** Environmental settings for high temperature treatment in the indoor growth chamber.

Step	Time	Duration(min)	Temperature(^0^C)	RH (%)	Light(μmol m-²s-¹)
1	6.30–7.00	30	27	75	330
2	7.00–7.30	30	30	75	460
3	7.30–8.00	30	35	70	580
4	8.00–8.30	30	38	70	580
5	8.30–14.30	360	38	70	580
6	14.30–15.30	60	35	70	580
7	15.30–16.30	60	30	70	580
8	16.30–17.30	60	27	75	460
9	17.30–18.30	60	24	75	330
10	18.30–6.30	720	24	75	0

### DNA Isolation

Leaves were sampled from 15 days old seedling to extract genomic DNA for molecular screening of high temperature tolerance amongst the genotypes. Total genomic DNA was extracted after crushing in liquid nitrogen in microfuge tubes using CTAB extraction buffer (100mM Tris-HCl pH 8, 20mM EDTA pH 8, 1.3M NaCl, 2% CTAB) and chloroform-Isoamyl alcohol extraction followed by RNAase treatment and ethanol precipitation [55]. Agarose gel electrophoresis was used to estimate DNA concentration and each sample was then diluted to approximately 30ng/μL.

### PCR amplification and visualization of markers linked to high temperature stress

DNA amplification reaction was performed in 20μl aliquot containing 1.5mM Tris HCL (pH 8.75), 50mM KCL, 2mM MgCl_2,_ 0.1% TrotonX-100, 200μM each of dATP, dCTP, dTTP, dGTP, 4pmole of each forward and reverse primers (Table 2), 1 unit of Taq polymerase and 30ng of *genomic* DNA. Amplification was done in a Programmable Thermal Cycler (Veriti, *App*lied BioSciences). The reaction mixture was first denatured for 4 min at 94°C and then subjected to 35 cycles of 1 min denaturation at 94°C, 1 min annealing at 55°C, 1 min extension at 72°C; and then a final extension for 10 mins at 72°C. Aliquots of 10μl of the products from PCR amplification were loaded in 2.5% agarose gel containing 0.8μg/ml Ethidium Bromide for electrophoresis in 1X TBE (pH 8.0). DNA ladder (50bp) was used for determination of size of amplicons. The gel was run at 60 volts (2.5V/cm) for 4 hrs and photographed using a Gel-Doc System (SynGene).

**Table 2:** Information on the selected linked molecular markers used for high temperature stress tolerance.

Sl. No.	Marker	Chr. No.	Primer sequences used for gene detection (5’– 3’)	Marker position	Expected Amplicon size (bp)	Reference
1	RM10346	1	GCTTGATCTGCCCTTGTTTCTTGG (F)	22.2cM	292	[56]
			AACTCGAGCGGCCTTCTCAGC (R)			
2	RM1209	1	AATGGAGCTCCTGACTCTAAAGC (F)	124.0cM	154	[56]
			TGCATCTCCTACAGAAACAAGG (R)			
3	RM570	3	AGAAATGGTGAAAGATGGTGCTACCG (F)	221.1cM	208	[33, 57, 58]
			CTGAATGTTCTTCAACTCCCAGTGC (R)			
4	RM3586	3	TCTTGATTGCTGGACCACATGC (F)	224.2cM	118	[34, 35, 58]
			TCGAGCTAGAAGACGACACACAGC (R)			
5	RM249	5	CAACTCCACTCCAGACTCAACTCC (F)	6.50cM	121	[59]
			GGTATGATGCCATGAAGGTCAGC (R)			
6	RM225	6	TATGTGGTTGGCTTGCCTAGTGG (F)	28.1–23.5cM	140	[36]
			TGCCCATATGGTCTGGATGTGC (R)			
7	RM314	6	CTAGCAGGAACTCCTTTCAGG (F)	20cM	118	[59]
			AACATTCCACACACACACGC (R)			
8	RM234	7	TTCAGCCAAGAACAGAACAGTGG (F)	107.2–113.8 cM	156	[60]
			CTTCTCTTCATCCTCCTCCTTGG (R)			
9	RM336	7	GTATCTTACAGAGAAACGGCATCG (F)	55.cM	154	[61]
			GGTTTGTTTCAGGTTCGTCTATCC (R)			
10	RM547	8	TTGTCAAGATCATCCTCGTAGC (F)	58.1cM	235	[33, 57, 58]
			GTCATTCTGCAACCTGAGATCC (R)			
11	RM205	9	CCTAAGAGGAGCCATCTAACAACTGG (F)	112.3–112.3 cM	122	[60]
			CTTGGATATACTGGCCCTTCACG (R)			
12	RM7364	9	TTTCGTGGATGGAGGGAGTACG (F)	1.7cM	204	[62]
			TGGCGACTTATGAGCGTTTGTAGG (R)			
13	RM219	9	CGTCGGATGATGTAAAGCCT (F)	0.8cM	202	[62]
			CATATCGGCATTCGCCTG (R)			
14	RM242	9	AAACACATGCTGCTGACACTTGC (F)	73.3cM	225	[33, 57, 58, 62]
			TTACTAGATTTACCACGGCCAACG (R)			
15	INDEL3	9	GGTTGCGACATTGGAGCCTTC (F)	1.5cM		[62]
			AATGCTTGGGTATGCTAGGTGAA (R)			
16	INDEL5	9	TCCTCGGAGATGTTTGACCTTG (F)	2.7cM		[62]
			CAGAAGGTGTACGCAACTCTTGT (R)			
17	RM228	10	TCTAACTCTGGCCATTAGTCCTTGG (F)	22.8cM	154	[59]
			AAGTAGACGAGGACGACGACAGG (R)			
18	RM209	11	ATATGAGTTGCTGTCGTGCG (F)	38.9cM	134	[59]
			CAACTTGCATCCTCCCCTCC (R)			
19	RM235	12	AAGCTAGGGCTAACGAACGAACG (F)	102.8cM	124	[33, 57, 58]
			TCTCCATCTCCATCTCCATCTCC (R)			
20	RM247	12	AAGGCGAACTGTCCTAGTGAAGC (F)	14.9–44.1 cM	131	[63]
			CAGGATGTTCTTGCCAAGTTGC (R)			

### Genetic Diversity and population structure

Data were scored on the basis of presence or absence of the alleles for each genotype-primer combination and entered into a binary data matrix as discrete variables. The number of alleles, allele frequency, gene diversity, heterozygosis and polymorphic information index (PIC) were estimated using the program PowerMarker Ver3.25 [64]. The data were analysed for possible population structure by using model based approach with STRUCTURE 2.3.4 software [65]. The project was run with 150,000 burn-in followed by 150,000 Markov Chain Monte Carlo (MCMC) replication with model parameter set of ‘possibility of admixture and allele frequency correlated’. Each K value was run for 10 times with K value varying from 1 to 10. The mean estimate of the log posterior probability of the data L (K) was plotted against the given K value in order to obtain optimum K value. Exact number of sub-population was identified using the maximal value of L (K). The model choice criterion to detect the most probable value of K was ∆K, an *ad hoc* quantity related to the second-order change of the log probability of data with respect to the number of clusters inferred by STRUCTURE [66]. The ΔK value as function of K estimated using Structure Harvester [67] has also shown a clear peak at the optimal K value. An unweighted neighbor joining un-rooted tree was constructed using the calculated dissimilarity index by using NEI coefficient [68] with bootstrap value of 1000 by using DARwin5 software [69]. distance matrix. The presence of molecular variance within and between the population structure was assessed through Analysis of molecular variance (AMOVA) by using GenAlEx 6.5 software [70]. F statistics including deviations from Hardy-Weinberg expectation across the whole population (F_IT_), deviation from Hardy-Weinberg expectation within a population (F_IS_) and correlation of alleles between subpopulation (F_ST_) was calculated. The hypothesis of the association of SSR markers with high temperature tolerance was tested using a general linear model (GLM) and mixed linear model (MLM) in the program TASSEL 5 [71].

## Results

### Phenotypic Screening of rice genotypes under field situation

**Fig 1:**
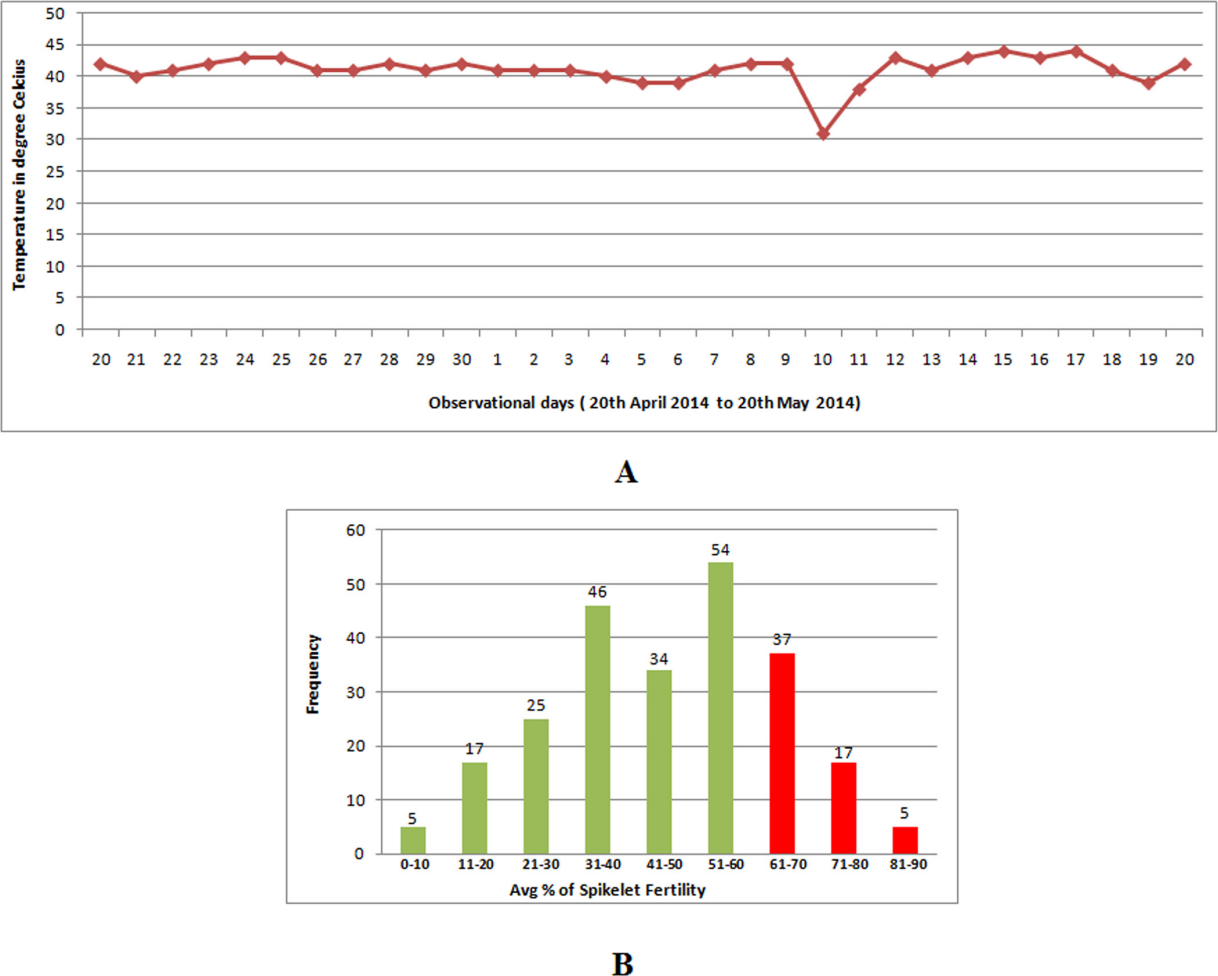
**(a)** Maximum temperature during the hottest time (April-May) in 2014 at National Rice Research Institute (NRRI), Cuttack, India, **(b)** 59 germplasm lines (red bars) were selected from 240 genotypes in the field selection in 2014 dry season for high temperature stress tolerance based on spikelet fertility from 240 germplasm lines under field screening during the hottest period (April-May month) of 2014, dry season.

A total of 240 upland and early duration rice germplasm lines were staggered grown during 2014 dry season at National Rice Research Institute (NRRI), Cuttack. These genotypes were exposed to the maximum temperature to coincide with anthesis during the hottest days of the year at the Institute site (Fig 1A). In the field evaluation study, 59 germplasm lines were selected from 240 genotypes based on spikelet fertility (SF) during the hottest period (April-May) of 2014 dry season (Fig 1B). The selected lines included 24 International Rice Research Institute tolerant lines and N22 (positive check) in it based on very high spikelet fertility (>60%) than the susceptible genotype Satyakrishna (Fig 1). The spikelet fertility of tested genotypes ranged from 7.6% (AC11209) to 88.6% (N22), while susceptible lines showed an average spikelet fertility of 15.3% (Fig 1; S1 Table).

### Phenotypic Screening of rice genotypes under control facility

The genotypes identified as tolerant in terms of high spikelet fertility in *ex situ* were screened again along with susceptible and tolerant checks under control facility (RGA-Cum-Phytotron) at NRRI, Cuttack following the protocol of Ye *et al*. [57] (Table 1). The phenotyping results exhibited a wide variation for percentage of spikelet fertility between 0.74 (Satyakrishna) and 64.29% (N22) under high temperature stress, whereas these genotypes showed very high fertility (>80%) in normal green house conditions (Table 3). Fourteen genotypes were observed to be sensitive to high temperature stress exhibiting a spikelet fertility range of 0.7–10%whilenormalunder*ex situ*. Twenty genotypes were weakly tolerant under high temperature stress with spikelet fertility of >10–20%, while all of them exhibited more than 80% spikelet fertility under normal condition. Eleven genotypes were moderately tolerant to the stress with spikelet fertility of >20–30%.Under temperature stress situation, seven genotypes behaved as tolerant type showing spikelet fertility of >30–40%. Eight genotypes namely CR3621-6-1-3-1-1, CR3820-2-1-5-1-2, CR3813-4-4-4-2-2, CR3820-4-5-3-1-3, AC.39890, AC.39973, AC.39790 and N22 showed spikelet fertility of >40% under high temperature stress and categorized as highly tolerant while these lines exhibited>80% under normal situation. Among all the genotypes studied, N22 alone was found to be very highly tolerant to high temperature stress showing >60% spikelet fertility while >80% fertility was observed under normal situation. The panicle exsertion varied with genotypes and ranged from well exserted (score1) to partially exserted (score7). Germplasm lines showed score 1 exhibiting well exserted panicle. Genotypes such as Satyakrishna and HHZ5-SAL10-DT3-Y2 scored more than 5 were observed to be susceptible to temperature stress.

**Table 3:** Details of SSR loci used for genotyping a set of 60 rice genotypes and their genetic diversity parameters.

Sl.No	Marker	Chr. No	SSR motif	Min.mol.wt	Max.mol.wt	No of alleles	Major allele frequency	Gene diversity	Heterozygosity	PIC value
1	RM10346	1	(AG)31	260	320	4	0.7833	0.3394	0.0000	0.2818
2	RM1209	1	(AG)14	155	170	2	0.5833	0.4861	0.0000	0.3680
3	RM570	3	(AG)15	250	255	2	0.6333	0.4644	0.0000	0.3566
4	RM3586	3	(GA)12	125	150	2	0.5333	0.4978	0.0000	0.3739
5	RM249	5	(AG)5A2(AG)14	130	145	2	0.8167	0.2994	0.0000	0.2546
6	RM225	6	(CT)18	125	145	3	0.8167	0.2994	0.0000	0.2546
7	RM314	6	(GT)8(CG)3(GT)5	110	130	2	0.6833	0.4328	0.0000	0.3391
8	RM234	7	(CT)25	140	165	3	0.5167	0.4994	0.0000	0.3747
9	RM336	7	(CTT)18	140	200	4	0.8667	0.2311	0.0000	0.2044
10	RM547	8	(ATT)19	200	250	4	0.5667	0.4911	0.0000	0.3705
11	RM205	9	(CT)25	100	150	5	0.7667	0.3578	0.0000	0.2938
12	RM7364	9	(CTAT)9	90	270	6	0.8833	0.2061	0.0000	0.1849
13	RM219	9	(CT)17	200	235	3	0.6000	0.4800	0.0000	0.3648
14	RM242	9	(CT)26	100	120	2	0.7833	0.3394	0.0000	0.2818
15	INDEL3	9	--	190	220	3	0.7833	0.3394	0.0000	0.2818
16	INDEL5	9	--	100	125	3	0.8333	0.2778	0.0000	0.2392
17	RM228	10	(CA)6(GA)36	115	155	4	0.8833	0.2061	0.1333	0.1849
18	RM209	11	(CT)18	140	165	3	0.7167	0.4061	0.0000	0.3236
19	RM235	12	(CT)24	100	140	3	0.7750	0.3488	0.0167	0.2879
20	RM247	12	(CT)16	135	160	3	0.5000	0.5000	1.0000	0.3750
	Mean						0.7163	0.3751	0.0575	0.2998

### Genotype-by-trait biplot analysis

**Fig 2:**
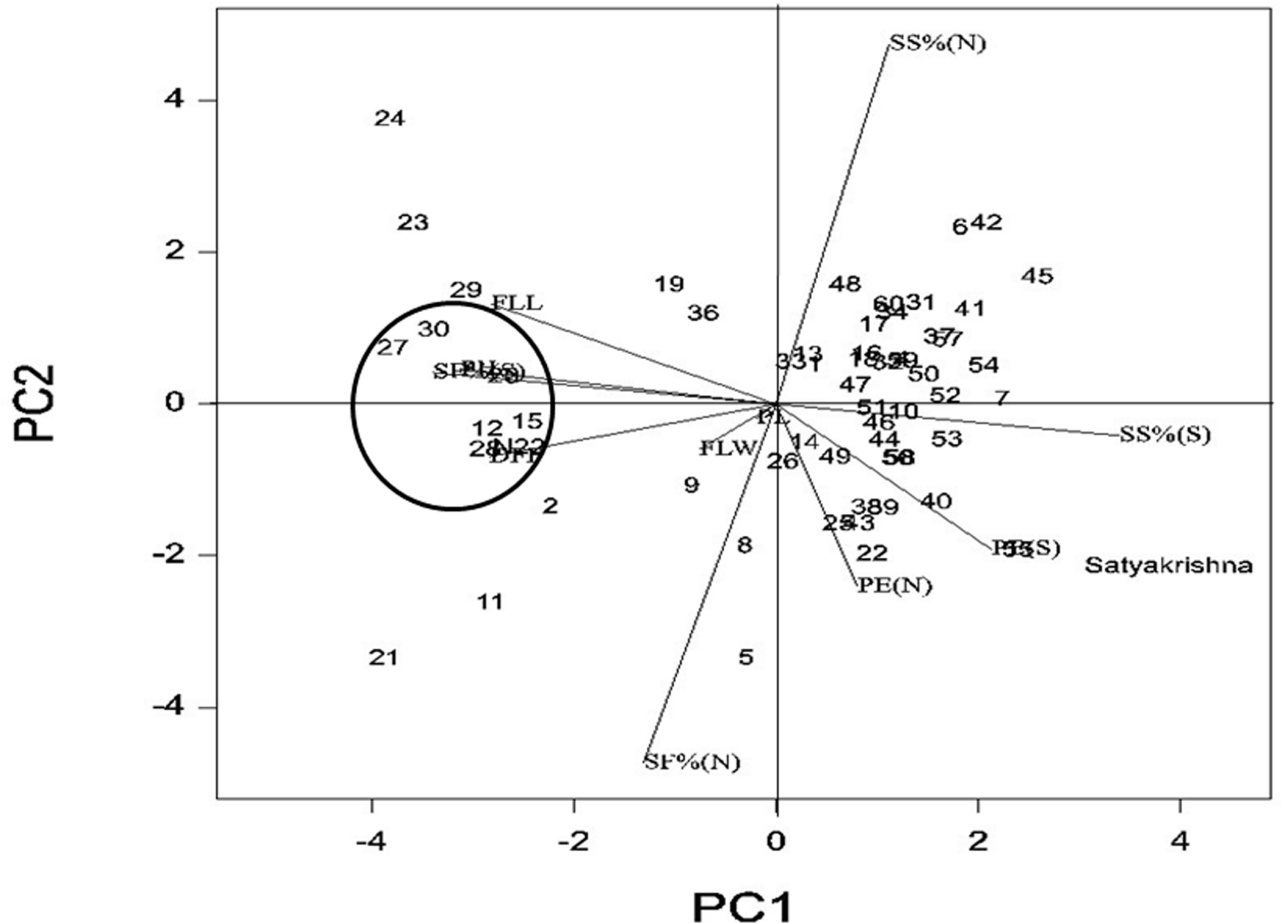
Genotype-by-trait biplot analysis of 60 genotypes for first two principal components. The numbers in the figure represent the serial number of the genotypes enlisted in Table 2. DFF-days to 50% flowering; PH-Plant height (cm.); FLL- Flag leaf length (cm.); FLB-Flag leaf breadth; PN-Panicles/plant; PL-Panicle length; SF(N)-Spikelet fertility under normal condition; SF(S)-Spikelet fertility under stress condition SS(N)-Spikelet sterility under normal condition; SS(S)-Spikelet sterility under stress condition; PE(N)-Panicle exsertion under normal condition; PE(N)-Panicle exsertion under stress condition.

The phenotyping data of 60 germplasm lines for 11 agro-morphologic traits were utilized to generate genotype-by-trait biplot graph (Fig 2) for analysis of the genotypes with first two principal components. The first principal component explained 64.68% of variation, while 2nd component exhibited 16.51% of the total variability. Among the 11 agro-morphologic traits studied, spikelet fertility % under normal condition contributed maximum towards diversity, followed by spikelet sterility under stress situation (Fig 2). The 1^st^ (top left) and 4^th^ (bottom left) quadrant contained 18 genotypes, which were tolerant to high temperature stress and exhibited high spikelet fertility under stress condition. The 4^th^ quarter accommodated genotypes which were more fertile under both the conditions as compared to 1^st^ quadrant and they were plotted away from origin. Important traits like spikelet sterility under normal and stress condition expressed higher values in the genotypes of 1^st^ and 4^th^ quarter justifying the presence of the highly tolerant genotypes in these two quarters. The 3^rd^quarter contains the intolerant genotypes to high temperature stress exhibiting higher estimates of traits like high sterility. The second quadrant genotypes were marginally better tolerant than the genotypes in 3^rd^ quadrant. The encircled area in the Fig 2 depicted five highly tolerant genotypes *viz*.,CR3820-2-1-5-1-2,CR3813-4-4-4-2-2, CR3820-4-5-5-3-1, AC39973 and AC39790 including N22 and one moderately tolerant accession AC11322, whereas, rest two highly tolerant genotypes AC39890 and CR3621-6-1-3-1-1 were marginally farther from the circle in the 1^st^ (top left) and 4^th^ (bottom left) quadrants respectively.

### Genetic diversity

A panel of 60 genotypes, comprising of landraces and breeding lines of tolerant and susceptible types were genotyped using two INDEL and 18 SSR linked markers for the trait. The details of the loci used for genotyping the set of 60 rice genotypes and their genetic diversity parameters obtained are presented in Table 4. A total of 63 alleles were amplified with the 20 co-dominant markers. The number of alleles per marker varied from 2 to 6 with an average of 3.15 per locus detecting the highest number of six alleles from RM 7364 in the set of 60 landraces and breeding lines. The mean PIC value was found to be 0.299 with minimum value of 0.185 (RM7364 and RM228) and maximum of 0.375 (RM247). The observed heterozygosity (H_o_) ranged between 0.0167 and 1.0, with an average H_o_ of 0.0575. Among the 20 markers tested, only three markers (RM228, RM235 and RM247) demonstrated the level of H_o_ more than zero, while others exhibited zero values. The expected heterozygosis or gene diversity (H_e_) ranged from 0.206 (RM247) to 0.5 (Indel4) followed by 0.499 (RM314) with an average of 0.3751. The major allele frequency of these high temperature stress tolerance linked polymorphic markers ranged from 0.500 to 0.883 with an average of 0.716 (Table 4).

**Table 4:** Comparison of 60 rice genotypes for panicle exesertion and spikelet fertility under normal and high temperature Stress condition.

Sl.No	Genotype name	Panicle Exsertion		Spikelet Fertility		Remarks	Sl.No	Genotype name	Panicle Exertion		Spikelet Fertility		Remarks
		Normal	Stress	Normal	Stress				Normal	Stress	Normal	Stress	
1	CR3622-7-3-1-1	1.00	3.00	82.9	29.66	MT	31	AC10984	1.00	3.00	81.1	12.22	WT
2	CR3820-4-5-3-1-3	1.00	3.00	88.8	33.28	T	32	AC10914	1.00	3.00	83.0	33.86	T
3	Satyakrishna	3.00	7.00	84.0	0.74	S	33	AC11261	1.00	3.00	83.9	31.53	T
4	CR143-2-2	1.00	3.00	83.0	22.39	MT	34	AC39843	1.00	3.00	81.7	21.02	MT
5	CR3825-2-1-2-2-3	3.00	3.00	90.7	17.32	WT	35	N22	1.00	3.00	87.5	64.29	HT
6	Sahabhagi	1.00	3.00	77.6	20.10	MT	36	Dular	1.00	1.00	83.1	18.06	WT
7	Satabdi	1.00	3.00	79.3	35.30	T	37	IR10C-137	1.00	3.00	81.3	8.90	S
8	Chandan	1.00	3.00	89.6	18.35	WT	38	IR10C-167	1.00	3.00	88.2	13.94	WT
9	BORO-4005	1.00	3.00	88.3	21.54	MT	39	IR10C-136	1.00	3.00	88.2	8.70	S
10	CR3825-2-1-2-2-4	1.00	3.00	85.1	14.44	WT	40	IR83142-B-36-B	1.00	3.00	87.8	12.46	WT
11	CR3621-6-1-3-1-1	1.00	3.00	93.5	41.59	HT	41	IR10C-108	1.00	3.00	81.1	10.23	WT
12	CR3820-2-1-5-1-2	1.00	3.00	86.3	40.08	HT	42	IR10C-161	1.00	3.00	77.9	9.63	S
13	CR2340-2	1.00	3.00	83.3	21.47	MT	43	HHZ17-Y16-Y3-Y1	1.00	3.00	89.3	16.22	WT
14	CRDhan-601	1.00	3.00	86.6	21.88	MT	44	IR10C-110	1.00	3.00	86.1	13.37	WT
15	CR3813-4-4-4-2-2	1.00	3.00	86.7	40.94	HT	45	HHZ8-SAL6-SAL3-SAL1	1.00	3.00	79.7	8.80	S
16	CR3826-8-3-2-1-1	1.00	3.00	82.2	9.40	S	46	IR10C-179	1.00	3.00	85.4	12.20	WT
17	CR2340-1	1.00	3.00	82.1	17.04	WT	47	HHZ11-DT7-SAL1-SAL1	1.00	3.00	83.3	11.18	WT
18	CR3622-7-3-2-2	1.00	3.00	82.7	7.90	S	48	IR10C-103	1.00	3.00	80.4	17.56	WT
19	CR3621-6-1-3-1-2	1.00	3.00	81.1	35.49	T	49	HHZ12-Y4-DT1-Y2	1.00	3.00	86.5	12.06	WT
20	CR3820-4-5-5-3-1	1.00	3.00	84.3	42.46	HT	50	IR10C-126	1.00	3.00	83.4	9.10	S
21	AC10976	1.00	1.00	97.5	27.06	MT	51	IR10G-103	1.00	3.00	84.5	20.28	MT
22	AC39975	3.00	5.00	84.4	15.07	WT	52	HHZ17-DT6-Y1-DT1	1.00	3.00	83.9	8.20	S
23	AC39890	1.00	1.00	81.1	42.99	HT	53	HHZ5-DT20-DT2-DT1	1.00	3.00	86.3	12.22	WT
24	AC11069	1.00	1.00	77.1	32.83	T	54	HHZ12-Y4-DT1-Y3	1.00	3.00	83.1	9.10	S
25	AC11311	1.00	3.00	88.6	8.40	S	55	HHZ5-SAL10-DT3-Y2	3.00	5.00	85.0	8.60	S
26	AC10925	1.00	3.00	87.0	34.12	T	56	IR83141-B-32-B	1.00	3.00	86.7	8.00	S
27	AC39973	1.00	1.00	85.5	41.16	HT	57	IR64197-3B-15-2	1.00	3.00	82.4	21.82	MT
28	AC11322	1.00	3.00	88.5	27.20	MT	58	HHZ5-SAL14-SAL2-Y2	1.00	3.00	87.2	18.24	WT
29	AC10994	1.00	3.00	82.0	17.05	WT	59	HHZ8-SAL6-SAL3-Y1	1.00	3.00	82.2	10.03	WT
30	AC39790	1.00	3.00	83.7	42.61	HT	60	IR10C-157	1.00	3.00	80.5	9.85	S

WT-weakly tolerant; MT-moderately tolerant; HT-Highly tolerant; S-susceptible.

### Cluster analysis

**Fig 3:**
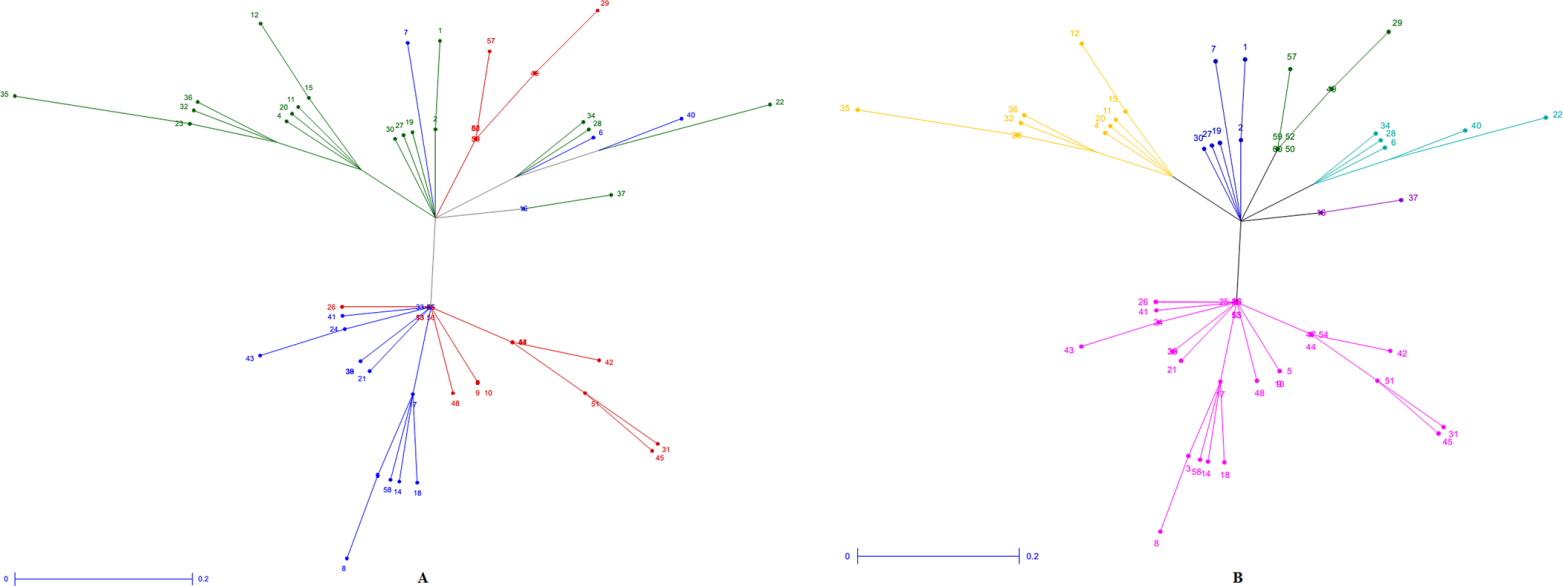
Unrooted tree using unweighted-neighbour joining method illustrating the genetic relationship among 60 genotypes based on 20 molecular markers colored on the basis of (A) individual clusters from origin point and (B) sub-populations obtained from structure analysis (SP1-red; SP2-blue and SP3-green). The numbers in the figure represent the serial number of the genotypes enlisted in Table 3.

The discrimination ability of the 20 closely linked markers for high temperature tolerance was determined by clustering the genotypes and by constructing the tree on the basis of amplification pattern with the markers. The unrooted tree was constructed by using unweighted-neighbour joining method (Fig 3). These polymorphic markers were distributed in 10 chromosomes and distinguished the genotypes into different groups. Four major and six minor clusters originated from the central point. The genotypes could be grouped into separate clusters exhibiting correspondent percentage of spikelet fertility (Fig 3A). The major cluster (yellow) grouped nine tolerant genotypes including established tolerant line N22. The genotypes namely N22, AC39890, AC10914, Dular, CR143-2-2, CR3820-4-5-5-3-1, CR3621-6-1-3-1-1, CR3813-4-4-4-2-2 and CR3820-2-1-5-1-2 included in this cluster showed high tolerance exhibiting spikelet fertility around 40% with an exception of two genotypes only. The genotypes IR10C-126, IR10C-157, HHZ8-SAL6-SAL3-Y1, HHZ17-DT6-Y1-DT1, HHZ12-Y4-DT1-Y2 and AC10994 possessing spikelet fertility 8–17% were grouped into separate cluster (green), including the genotype IR64197-3B-15-2, which exhibited 21.82% spikelet fertility. Germplasm lines AC39843, AC11322, Sahabhagidhan, IR83142-B-36-B and AC39975 were grouped together as single cluster and genotypes CR3826-8-3-2-1-1 and IR10C-137 formed a separate cluster. Thirty one genotypes formed one major cluster which was again divided into sub clusters. One sub cluster grouped the genotypes HHZ11-DT7-SAL1-SAL1, HHZ12-Y4-DT1-Y3, IR10C-110, IR10C-161, IR10G-103, AC10984 and HHZ8-SAL6-SAL3-SAL1 exhibiting spikelet fertility from 8.8% to 13.37%. The genotypes CR3825-2-1-2-2-3 (SF 17.31%), Boro-4005 (SF 21.54%) and CR3825-2-1-2-2-4 (SF 14.44%) formed one sub-cluster. Similarly, the genotypes namely HHZ5-SAL10-DT3-Y2, CR3622-7-3-2-2, CR Dhan 601, HHZ5-SAL14-SAL2-Y2, CR2340-1, Satyakrishna and Chandan formed a distinct sub cluster.

### Population structure

**Fig 4:**
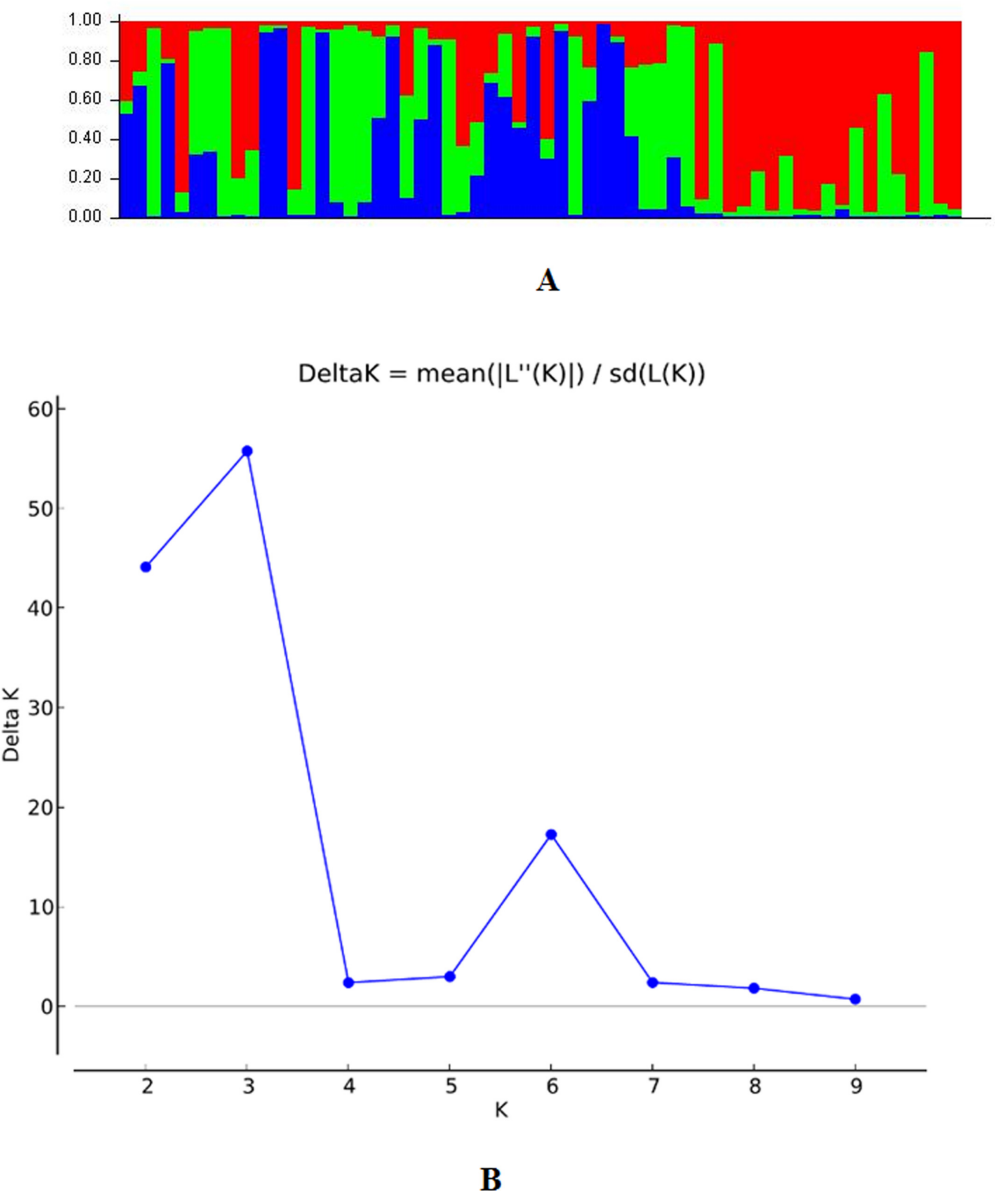
(a) Population structure of a panel of 60 genotypes based on 20 molecular markers (K = 3) and (b) Graph of estimated membership fraction for K = 3. The maximum of adhoc measure ΔK determined by structure harvester was found to be K = 3, which indicated that the entire population can be grouped into two sub-groups (SP1, SP2 and SP3).

**Fig 5:**
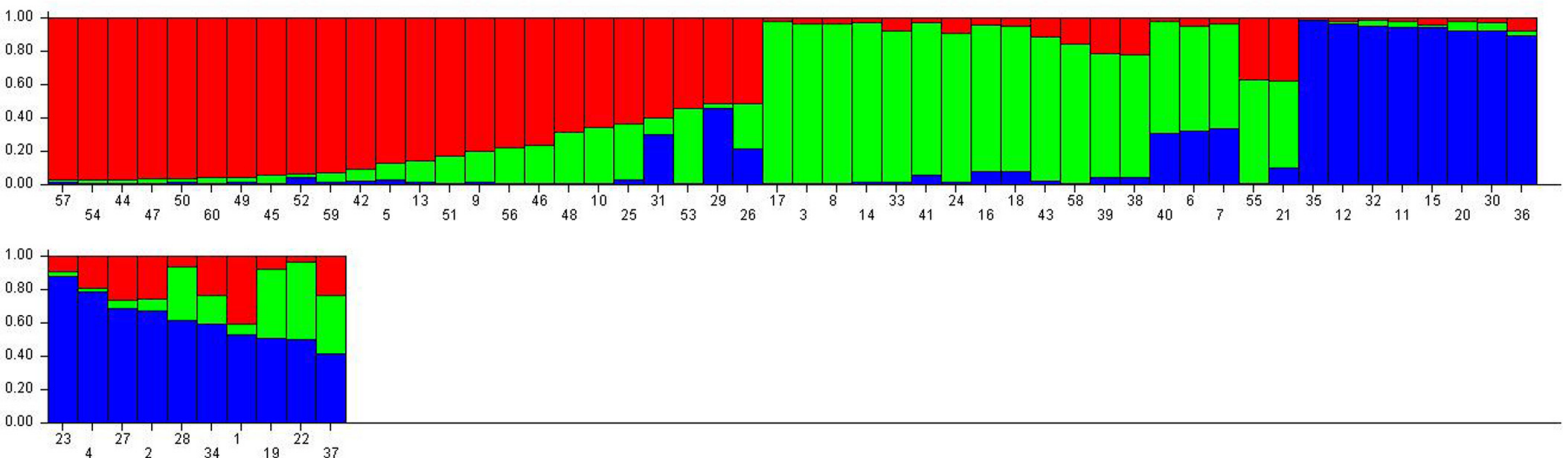
Population structure of a panel of 60 landraces and breeding lines arranged based on inferred ancestry. The membership fractions, the genotypes with the probability of ≥ 80% were assigned to corresponding subgroups with others categorized as admixture.

The population panel for high temperature stress tolerance was analyzed by employing STRUCTURE software utilizing the Bayesian clustering approach that divided the population into three sub-populations (Figs 4 and 5). The assumed values of probable sub-populations (K) were ascertained by choosing higher ∆K value, an *ad hoc* quantity related to the second order change of the log probability of data with respect to the number of clusters inferred by Structure [66]. As per the Evano table output, the K = 3 was observed to be the best due to high ∆K peak value of 55.8 among the assumed K (Fig 4B). The sub-population 1 (SP1) contained 15 pure and 9 admixture genotypes, accommodating weakly tolerant and susceptible genotypes to high temperature stress. In the structure analysis, pure and admixture categorization was made with 18 pure and 7 admixture types from a total of 25 germplasm lines in sub-population2 representing mainly weakly and moderately tolerant types (Figs 4 and 5; Table 5). The third sub-population can be categorized as tolerant group, it possessed tolerant and highly tolerant germplasm lines with 9 pure and 9 admixture ones. The fixation index values (F_ST_) of the sub-populations were estimated to be 0.521, 0.406 and 0.325 for SP1, SP2 and SP3, respectively. Further, output of the program detected a lower value of alpha (α = 0.139) in the population set. The distribution pattern of α value in the population and distribution of F_ST_ values in the sub-populations are depicted in S1 Fig.

**Table 5:** Population structure group of accessions based on inferred ancestry values.

Sl.No.	Genotypes	Inferred ancestry			Structure group	Pedigree	Origin	Reaction to high temperature stress
		Q1	Q2	Q3				
1	CR3622-7-3-1-1	0.404	0.065	0.531	SP3	Naveen/NLR33892//NLR 9692–96	India	MT
2	CR3820-4-5-3-1-3	0.255	0.071	0.673	SP3	AC.38562/Ratna//Niraj	India	MT
3	Satyakrishna	0.032	0.960	0.008	SP2	Doubled haploid of PHB71	India	S
4	CR 143-2-2	0.189	0.023	0.788	SP3	Bala/Lalnakanda	India	MT
5	CR3825-2-1-2-2-3	0.868	0.101	0.031	SP1	IR64/NLR9672-96	India	WT
6	Sahabhagidhan	0.045	0.629	0.326	SP2	IR 74371-70-1-1-CRR-1/Way Rarem	India	MT
7	Satabdi	0.034	0.629	0.337	SP2	CR 10-114/CR 10–115	India	MT
8	Chandan	0.031	0.957	0.011	SP2	Gamma ray mutant of China 45	India	WT
9	BORO 40059	0.797	0.186	0.017	SP1	Krishna Hamsa/IR73933-8-CR-2-2-1	India	MT
	(CR3618-4-3-1-1-1)							
10	CR3825-2-1-2-2-4	0.653	0.334	0.013	SP1	IR64/NLR9672-96	India	WT
11	CR3621-6-1-3-1-1	0.016	0.036	0.947	SP3	IR64/Dandi//Lalat	India	HT
12	CR3820-2-1-5-1-2	0.019	0.012	0.969	SP3	AC.38562/Ratna//Niraj	India	HT
13	CR2340-2	0.856	0.128	0.017	SP1	IR31238-350-3-2-1 /IR41054-102-2-3-2	India	MT
14	CR Dhan 601	0.023	0.957	0.020	SP2	IR64/Jaya	India	MT
15	CR3813-4-4-4-2-2	0.037	0.020	0.943	SP3	CR780-1937-1-3/DRR1702	India	HT
16	CR3826-8-3-2-1-1	0.037	0.878	0.085	SP2	Satabdi/NLR 9672-96//Lalat	India	S
17	CR2340-1	0.017	0.975	0.008	SP2	IR31238-350-3-2-1 /IR41054-102-2-3-2	India	WT
18	CR3622-7-3-2-2	0.044	0.871	0.085	SP2	Naveen/NLR33892//NLR 9692–96	India	S
19	CR3621-6-1-3-1-2	0.072	0.419	0.509	SP3	IR64/Dandi//Lalat	India	MT
20	CR3820-4-5-5-3-1	0.018	0.054	0.928	SP3	CRG 1190-1/CR 898	India	HT
21	AC10976	0.376	0.519	0.104	SP2	Landrace,Odisha	India	MT
22	AC39975	0.033	0.460	0.507	SP3	Landrace,Odisha	India	WT
23	AC39890	0.090	0.027	0.883	SP3	Landrace,Odisha	India	HT
24	AC11069	0.088	0.894	0.018	SP2	Landrace,Odisha	India	MT
25	AC11311	0.629	0.337	0.034	SP1	Landrace,Odisha	India	S
26	AC10925	0.509	0.271	0.220	SP1	Landrace,Odisha	India	MT
27	AC39973	0.264	0.046	0.691	SP3	Landrace,Odisha	India	HT
28	AC11322	0.064	0.318	0.618	SP3	Landrace,Odisha	India	MT
29	AC10994	0.514	0.026	0.460	SP1	Landrace,Odisha	India	WT
30	AC39790	0.028	0.044	0.928	SP3	Landrace,Odisha	India	HT
31	AC10984	0.599	0.100	0.301	SP1	Landrace,Odisha	India	WT
32	AC10914	0.011	0.037	0.952	SP3	Landrace,Odisha	India	MT
33	AC11261	0.072	0.913	0.014	SP2	Landrace,Odisha	India	MT
34	AC39843	0.235	0.171	0.593	SP3	Landrace,Odisha	India	MT
35	N22	0.008	0.006	0.986	SP3	Selection from landrace of Nagina, Azamgarh, India	India	HT
36	Dular	0.078	0.023	0.899	SP3	Selection from landrace	India	WT
37	IR10C-137	0.229	0.356	0.415	SP3	Super basmati/ IR2006-P12-12-2-2	IRRI	S
38	IR10C-167	0.218	0.736	0.046	SP2	CT 6946-9-1-2-M-1P/IRRI 123	IRRI	WT
39	IR10C-136	0.214	0.739	0.047	SP2	Super basmati/ IR2006-P12-12-2-2	IRRI	S
40	IR83142-B-36-B	0.021	0.671	0.307	SP2	IR06G103/ IR06G113	IRRI	WT
41	IR10C-108	0.025	0.911	0.064	SP2	IR 6/DSA 77	IRRI	WT
42	IR10C-161	0.905	0.069	0.026	SP1	CT 6946-9-1-2-M-1P/IRRI 123	IRRI	S
43	HHZ17-Y16-Y3-Y1	0.114	0.862	0.024	SP2	HUANG-HUA-ZHAN*2/CDR 22	IRRI	WT
44	IR10C-110	0.966	0.026	0.008	SP1	IR 6/DSA 77	IRRI	WT
45	HHZ8-SAL6-SAL3-SAL1	0.939	0.049	0.012	SP1	HUANG-HUA-ZHAN*2/PHALGUNA	IRRI	S
46	IR10C-179	0.758	0.231	0.011	SP1	IR 2344-P1 PB-9-3-2B/IR 64	IRRI	WT
47	HHZ11-DT7-SAL1-SAL1	0.964	0.025	0.011	SP1	HUANG-HUA-ZHAN*2/IR 64	IRRI	WT
48	IR10C-103	0.681	0.305	0.015	SP1	GIZA 178/IR 2006-P12-12-2-2	IRRI	WT
49	HHZ12-Y4-DT1-Y2	0.953	0.026	0.021	SP1	HUANG-HUA-ZHAN*2/TE QING	IRRI	WT
50	IR10C-126	0.963	0.019	0.018	SP1	IR 64/ IR 2006-P12-12-2-2	IRRI	S
51	IR10G-103	0.822	0.168	0.009	SP1	IR06G103/ IR06G116	IRRI	MT
52	HHZ17-DT6-Y1-DT1	0.933	0.023	0.043	SP1	HUANG-HUA-ZHAN*2/CDR 22	IRRI	S
53	HHZ5-DT20-DT2-DT1	0.536	0.455	0.009	SP1	HUANG-HUA-ZHAN*2/OM 1723	IRRI	WT
54	HHZ12-Y4-DT1-Y3	0.967	0.025	0.008	SP1	HUANG-HUA-ZHAN*2/TE QING	IRRI	S
55	HHZ5-SAL10-DT3-Y2	0.369	0.620	0.011	SP2	HUANG-HUA-ZHAN*2/OM 1723	IRRI	S
56	IR83141-B-32-B	0.775	0.214	0.011	SP1	IR06G103/ IR06G112	IRRI	S
57	IR64197-3B-15-2	0.969	0.014	0.017	SP1	IR 42598-B-B-B-B-12/NONA BOKRA	IRRI	MT
58	HHZ5-SAL14-SAL2-Y2	0.157	0.833	0.010	SP2	HUANG-HUA-ZHAN*2/OM 1723	IRRI	WT
59	HHZ8-SAL6-SAL3-Y1	0.927	0.052	0.021	SP1	HUANG-HUA-ZHAN*2/PHALGUNA	IRRI	WT
60	IR10C-157	0.956	0.033	0.011	SP1	CT 6946-9-1-2-M-1P/IR 64	IRRI	S

WT-weakly tolerant; MT-moderately tolerant; HT-Highly tolerant; S-susceptible.

### Analysis of molecular variance (AMOVA)

The three populations generated from structure analysis were analyzed for genetic variation among and within the clusters using AMOVA (Table 6). From the analysis, 25% of variation was observed between population, 61% among individuals and 14% within individuals observed in the population. Wright’s F statistic was estimated to determine deviation of Hardy-Weinberg expectation in the population. The F_IS_ for all the 20 marker loci was 0.815, while F_IT_was 0.862 across the clusters. Pairwise F_ST_ values showed significant differentiation among all the pairs of sub-populations ranging from 0.192 to 0.296 suggesting that all the three groups were significantly different from each other. The F_ST_ values and their distribution pattern show clear differentiation of sub populations from each other (S1 Fig).

**Table 6:** AMOVA between sub-populations and germplasm lines and fixation indices (GenAlEx 6.5 software).

Source of variations	df	Sum of squares	MS	Est. Var.	%of variation
Among Populations	2	94.151	47.076	1.046	25%
Among Individuals within populations	57	321.507	5.640	2.533	61%
Within Individuals	60	34.500	0.575	0.575	14%
Total	119	450.158		4.154	100%
F-Statistics	Value	P(rand > = data)			
Fst	0.252	0.001			
Fis	0.815	0.001			
Fit	0.862	0.001			

### Association of marker alleles with phenotypic traits

The marker-trait associations for all the traits were calculated using GLM and MLM (Q+K) model of TASSLE5 software (S2 Table). The squared allele frequency correlation (*r*^*2*^) values ranged from 0.066 to 0.288 with an average of 0.125 by using GLM, whereas the average reduced to 0.016 by using MLM analysis. Among total of 220 comparisons, 10 were found to be significant at *p*< 0.01 and 36 were significant at *p* < 0.05 by using GLM, whereas MLM analysis showed only 10 comparisons to be significant at *p* < 0.05 (Table 7). The markers showing significant associations were within 20cM range with an exception of few markers like RM547 and RM336. However, the marker RM7365 reported to be tightly linked to high temperature stress tolerance with 0.8cM position in earlier bi-parental mapping did not show significant association in the present study. The markers located more than 100cM distance did not show association at *p <0*.*01*, as in the case of RM235 and RM234 located on chromosome 12 and 7, respectively were not significant at *r*^*2*^> 0.10 with *p*< 0.05.

**Table 7:** Association of marker alleles with phenotypic traits under high temperature stress using GLM and MLM analysis in 60 rice genotypes.

Trait	Marker name	GLM			MLM		
		F value	P value	R^2^	F value	P value	R^2^
Plant height	RM570	8.85585	0.00425	0.13246	-	-	-
Plant height	RM249	6.17135	0.01589	0.09617	-	-	-
Plant height	RM547	6.71632	0.01207	0.10378	-	-	-
Plant height	INDEL5	8.18195	0.00587	0.12363	-	-	-
Plant height	RM1209	0.27241	0.60371	0.00467	-	-	-
Plant height	RM242	0.94893	0.33404	0.0161	-	-	-
Days to 50% flowering	RM3586	4.16118	0.04592	0.06694	-	-	-
Days to 50% flowering	RM225	5.23377	0.02582	0.08277	-	-	-
Days to 50% flowering	RM336	6.92799	0.01086	0.1067	-	-	-
Days to 50% flowering	RM547	17.76243	8.86E-05	0.23445	-	-	-
Days to 50% flowering	INDEL3	3.33248	0.07307	0.05433	4.44623	0.03931	0.07536
Panicle length	RM3586	4.33391	0.04178	0.06953	-	-	-
Panicle length	RM242	6.799	0.01158	0.10492	4.79804	0.03253	0.08751
Panicle length	INDEL3	8.70586	0.00457	0.13051	6.14541	0.0161	0.11208
Flag leaf length	RM225	7.46328	0.00833	0.11401	-	-	-
Flag leaf length	RM205	4.86184	0.03144	0.07734	5.41615	0.02346	0.0918
Flag leaf length	RM547	14.28096	3.74E-04	0.19758	4.72166	0.03388	0.08003
Flag leaf length	RM336	1.80323	0.18455	0.03015	-	-	-
Flag leaf width	RM247	4.12795	0.04677	0.06644	5.79824	0.01924	0.09828
Flag leaf width	INDEL5	4.39623	0.04039	0.07046	-	-	-
Panicle emergence (normal)	RM228	7.88738	0.00677	0.11971	7.06245	0.01015	0.1197
Panicle emergence (stress)	RM249	5.07145	0.02812	0.08041	-	-	-
Panicle emergence(stress)	RM228	5.91254	0.01814	0.09251	-	-	-
Spikelet fertility (normal)	RM314	6.97862	0.01059	0.1074	4.37586	0.04084	0.07417
Spikelet fertility (stress)	RM3586	4.22441	0.04436	0.06789	-	-	-
Spikelet fertility (stress)	RM249	10.06263	0.00242	0.14784	-	-	-
Spikelet fertility (stress)	RM225	13.34653	5.58E-04	0.18707	-	-	-
Spikelet fertility (stress)	RM336	10.24155	0.00223	0.15008	-	-	-
Spikelet fertility (stress)	RM547	23.49859	9.73E-06	0.28833	5.22249	0.02597	0.08852
Spikelet sterility (normal)	RM314	5.04848	0.02847	0.08007	-	-	-
Spikelet sterility (normal)	RM219	4.66249	0.03498	0.07441	-	-	-
Spikelet sterility (stress)	RM3586	4.22441	0.04436	0.06789	-	-	-
Spikelet sterility (stress)	RM249	10.06263	0.00242	0.14784	-	-	-
Spikelet sterility (stress)	RM225	13.34653	5.58E-04	0.18707	-	-	-
Spikelet sterility (stress)	RM336	10.24155	0.00223	0.15008	-	-	-
Spikelet sterility (stress)	RM547	23.49859	9.73E-06	0.28833	5.22249	0.02597	0.08852

**Fig 6:**
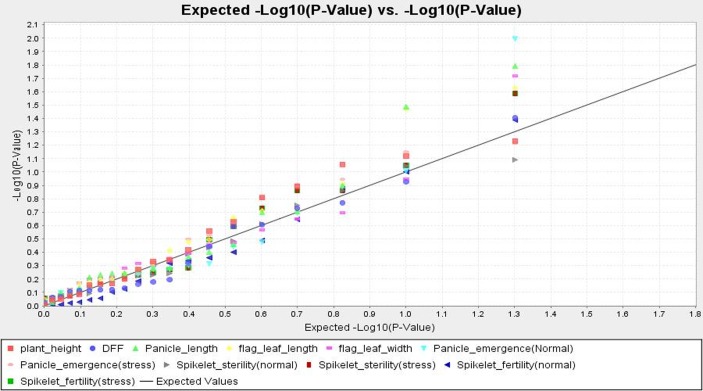
Quantile-Quantile (QQ) plot and distribution of marker-trait association from MLM analysis.

Considering the GLM statistics, RM314, RM 3586, RM249, RM225, RM336 and RM547 could be associated with spikelet fertility percentage, with higher F value and lower P value indicating a positive association with high temperature tolerance. Further, to make the association robust by considering the kinship value, MLM analysis exhibited a strong marker-trait association with phenotypic variance of 7.42% with RM314 and 8.85% with RM547 for trait spikelet fertility under normal and stress condition, respectively. It was further evidenced from TASSEL analysis that the marker RM228 associated with panicle emergence; RM242 and INDEL3 with panicle length; RM 247 with flag leaf width; RM205, RM547 with flag leaf length; INDEL3 with days to 50% flowering which was also evident from cluster analysis. INDEL3 associated with days to 50% flowering and panicle length, whereas RM547 showed strong association with flag leaf length, spikelet fertility and sterility under stress condition. The QQ plot showed significant association of markers for spikelet fertility/ sterility under stress, panicle emergence under stress, panicle length and plant height (Fig 6).

## Discussion

High temperature stress is posing as an important yield limiting abiotic factor in dry season rice of many Asiatic countries. It is causing significant yield loss in case of delayed dry season rice cultivation in sub-tropical countries due to changing climate. Hence, genetic diversity for this trait is the pre-requisite step for developing high yielding temperature stress tolerant rice varieties suitable for dry season cultivation. In our field screening along with concurrent controlled screening methods, we could identify eight highly tolerant genotypes to high temperature stress with >40% spikelet fertility under stress condition. The genotypes were classified into four classes basing on spikelet fertility. Clustering based on molecular markers and genotype-trait biplot analysis showed almost similar pattern of grouping to that of the spikelet fertility phenotyping group (Figs 2 and 3). The tolerant genotypes were grouped into many sub-groups, which might be due to the presence of different stress tolerant gene(s)/QTL(s) and their possible combinations in the landraces and breeding lines. Hence, the population structure estimation in the germplasm set is most important. Earlier studies also indicated screening and identification of heat tolerant genotypes in rice [14,17,20,72,73]. N22, an upland cultivar of India was observed to be most tolerant genotype showing high spikelet fertility under field and control screening facility. Similar results were also obtained in earlier screening for high temperature stress tolerance [21].

The principal component analysis placed the tolerant genotypes in 1^st^ and 4^th^ quadrant of the biplot on the basis of eleven agro-morphologic traits, where highly tolerant genotypes were highlighted in a circle (Fig 2). The 60 genotypes were classified into tolerant and non-tolerant with most of the tolerant types in the left side of center. Hence, there is a variation for the trait with respect to temperature tolerance. The unrooted tree, discriminated the highly tolerant and tolerant classes on the basis of 20 temperature stress linked molecular markers. The genotypes could be grouped into separate clusters almost matching to the percentage of spikelet fertility. Various classes of genotypes obtained based on temperature tolerance linked markers indicated the presence of many genes/QTLs in the studied genotypes. In our study, 20 high temperature stress tolerance linked molecular markers produced 63 alleles with average gene diversity of 0.3751 and PIC value of 0.2998. The low to moderate value of alleles, gene diversity and PIC might be due to the use of limited numbers of linked markers for a single phenotypic trait with the exclusion of spurious bands from the genotyping analysis. As the diversity estimation is for multi-locus alleles conferring diversity with respect to a high temperature tolerance trait, these parameters indicate higher genetic diversity in the population for the trait. Similar results were observed in earlier studies in rice diversity using single trait linked markers [74,75]. In a study of 40 accessions from Pakistan [75] indicated slightly lower genetic diversity with average PIC values of 0.38 with an average of 2.75 alleles per locus. Our results on genetic diversity of high temperature stress tolerance are in conformity with the earlier studies indicating moderate diversity parameters estimate [74–79]. However, several earlier reports also suggested very rich values of the genetic diversity for agro-morphologic traits in various rice populations [50,80,81].

The existence of linkage disequilibrium between a molecular marker and an unknown locus regulating a particular phenotype can further lead to establishment of association of the specific haplotype/ marker alleles with that particular phenotype in a population. The associated marker alleles can be used in molecular breeding program for improving other genotypes lacking the particular trait. The marker should be tightly linked to the locus controlling the particular phenotype for detection of marker-trait association. In our study, marker-trait association was much more precise as the materials showed different classes of phenotypes for high temperature stress tolerance trait of rice. Structured population and relatedness characteristics among the set of genotypes suggested that the population structure and kinship in conducting population based association mapping in rice germplasm should be performed. The phenotypic evaluation clearly differentiated the study materials into different groups, indicating their heterogeneity towards high temperature stress tolerance (Figs 2–4).This favoured the presence of linkage disequilibrium and increased the chance of detecting marker-trait association. Similar results showing the potential value of germplasm in heterogeneous collections had detected marker-phenotypic trait association [35,50,64,77,82,83,84].

High temperature tolerance in rice has gained much attention due to increasing temperature stress, particularly in dry season rice cultivation of sub-tropical countries. The application of association mapping for this trait will be helpful in deciding the molecular markers to be used in pyramiding the multiple QTLs responsible for the trait. Therefore, diverse populations are needed for association mapping of the trait. STRUCTURE has grouped the population into three sub-populations, indicating the suitability of the selected population for phenotype-trait association study. Further, output of the program detected a lower value of alpha (α = 0.139) suggesting most of the landraces in each sub-population had a common primary ancestor with few admix individuals. The inferred ancestry indicated that many landraces possess a partial allele from an ancestry, which might probably be due to complex intercrossing among germplasm from diverse background experiencing strong selection pressure for the trait. Similar reason has also been reported by earlier workers [50,85].

High *r*^*2*^ values were detected for markers RM547, RM228, RM247, RM205, RM indel3, RM242 and RM314 by both GLM and MLM TASSEL analysis for spikelet fertility and other phenotypic traits of tolerant accessions. This suggests that, the observed marker-trait association resulted from a possibly multiple introgressions from the landraces possessing different QTLs which ultimately showed a higher level of tolerance. Of the 220 comparisons, only 10 were significant with *r*^*2*^> 0.10 at *p*< 0.01. Thirty six comparisons were significant at p<0.05 (Table 7). The markers showing significant associations fall within 20cM range with an exception of the few markers like RM547 and RM336. Similar results were also earlier described by Zhao *et al*. [50]. Besides spikelet fertility (stress), RM 547 was detected to be strongly associated with flag leaf length. This indicated that one marker could show association with other regions of the rice genome also. However, the genetic and physical distance between a marker and the genes controlling quantitative traits might be quite important for detecting association. These linked marker positions and other quantitative trait might also show a pleiotropic effect or may be located very closely. Similar multiple quantitative traits association with molecular markers have been detected and reported earlier [82,86]. One marker allele namely RM547 showed strong association with spikelet fertility detected by both GLM and MLM analysis can be used for selecting high temperature stress tolerant lines in rice (Table 7). In the present marker-trait association study, a strong association was detected by MLM and GLM model of TASSEL analysis for marker RM314 with spikelet fertility (normal); RM228 for panicle emergence; RM205 and RM547 for flag leaf length; RM247 for flag leaf width; RM 242 and INDEL3 for panicle length and INDEL3 for days to 50% flowering. Earlier reports using various mapping populations have reported many high temperature tolerant QTLs on chromosome 6,8,9,10 and 12 [33,57,59,60, 62, 63]. Also, our principal component study revealed higher estimates for these phenotypic traits in the tolerant lines present in the encircled area of the biplot analysis (Fig 2). Hence, we may use these six associated markers in marker-assisted breeding for improvement of these traits by which indirectly tolerance for high temperature will increase. Thus, the strongly associated marker RM547 with spikelet fertility under stress and the markers indirectly controlling the high temperature stress tolerance like RM228, RM205, RM247, RM242, INDEL3 and RM314 can be used in marker-assisted high temperature stress tolerance breeding program.

## Conclusion

The phenotypic screening study for high temperature stress tolerance has classified the set of genotypes into four classes. A moderate level of genetic base of the population could be inferred from the genetic diversity analysis. The population panel for the trait has been categorized into three sub-populations by STRUCTURE software. It suggested that most of the landraces in each sub-population had a common primary ancestor with few admix individuals. A set of landraces and breeding lines from a large collection of germplasm materials showed the advantage of many QTLs in the set of materials representing in almost the whole of the genome for the trait. Application of association mapping will increase the utility of more number of markers detected from this study in future molecular breeding programmes aimed at improving this trait. This type of mapping can be useful and a suitable and dependable alternative for linkage mapping, making marker-assisted selection exercise more effective.

## Supporting Information

S1 Fig Distribution pattern of α value in the population and distribution of F_ST_ values in the sub-populations. (DOCX)

S1 Table Germplasm lines used in field screening for high temperature stress tolerance and their spikelet fertility under field screening. (DOCX)

S2 Table Dataset (60 accessions x 20 Markers) used for analysis in TASSEL 5. (XLSX)

## References

[1] Food and Agricultural Organisation. FAOSTAT Database Rome: Food and Agricultural Organization. Yearbook; 2013.

[2] Matsushima S, Ikewada H, Maeda A, Honda S, Niki H. Studies on rice cultivation in the tropics. In: Yielding and ripening responses of the rice plant to the extremely hot and dry climate in Sudan. Jpn. J. Trop. Agric.1982; 26: 19–25.

[3] Osada A, Sasiprapa V, Rahong M, Dhammanuvong S, Chakrabandho H. Abnormal occurrence of empty grains of indica rice plants in the dry hot season in Thailand. Proc. Crop Sci. Soc. Jpn. 1973;42: 103–109.

[4] Li J, Xiao JH, Grandillo S, Jiang LY, Wan YZ, Deng QY, et al QTL detection for rice grain quality traits using an interspecific backcross population derived from cultivated Asian (*O*. *sativa* L.) and African (*O*. *glaberrima* S.) rice. Genome.2004; 47(4):697–704. 10.1139/g04-029 15284874

[5] Xia MY, Qi HX. Effects of high temperature on the seed setting percent of hybrid rice bred with four male sterile lines. Hubei Agric. Sci. 2004;2: 21–22.

[6] Yang Y, Qi M, Mei C. Endogenous salicylic acid protects rice plants from oxidative damage caused by aging as well as biotic and abiotic stress. The Plant Journal.2004;40:909–919. 15584956 10.1111/j.1365-313X.2004.02267.x

[7] Root TL, Price JT, Hall KR, Schneider SH, Rosenzweigk C, Pounds JA. Fingerprints of global warming on wild animals and plants.Nature.2003; 421(2):57–60. 12511952 10.1038/nature01333

[8] Meehl GA, Washington WM, Collins WD, Arblaster JM, Hu A, Buja LE, et al How Much More Global Warming and Sea Level Rise. Science. 2005;18: 1769–1772, 10.1126/science.110666 15774757

[9] Vuuren DPV, Meinshausenc M, Plattnerd GK, Joose F, Strassmanne KM, Smithg SJ, et al Temperature increase of 21st century mitigation scenarios. Proc. Natl. Acad. Sci.2008;105 (40): 15258–15262. 10.1073/pnas.0711129105 18838680 PMC2562414

[10] Peng SB, Huang JL, Sheehy JE, Laza RC, Visperas RM, Zhong XH, et al Rice yields decline with higher night temperature from global warming. Proc. Natl. Acad. Sci. 2004;101:9971–9975. 15226500 10.1073/pnas.0403720101 PMC454199

[11] Sato K, Inaba K, Tosawa M High temperature injury of ripening in rice plant. The effects of high temperature treatment at different stages of panicle development on the ripening. Proceedings of Crop Science Society of Japan.1973; 42:207–13.

[12] Satake T, Yoshida S. High temperature induced sterility in indica rice at flowering. Jpn. J. Crop Sci.1978; 47: 6–17.

[13] Yoshida S, Satake T, Mackill DJ. High temperature stress in rice (review). IRRI Res. Paper Series.1981; 67: 5.

[14] Jagadish S, Crauford PQ, Wheeler TR. High temperature stress and spikelet fertility in rice (*Oryza sativa* L.). J. Exp Bot. 2007; 58: 1627–35. 17431025 10.1093/jxb/erm003

[15] Mackill DJ, Coffman WR, Rutger JN. Pollen shedding and combining ability for high temperature tolerance in rice. Crop Sci. 1982; 22: 730–733.

[16] Matsui T, Omasa K, Horie T. High temperature induced spikelet sterility of japonica rice at flowering in relation to air humidity and wind velocity conditions. Jpn.J. Crop Sci. 1997; 66: 449–455.

[17] Matsui T, Omasa K, Horie T. The differences in sterility due to high temperatures during the flowering period among japonica rice varieties. Plant Production Science. 4: 90–93.23.

[18] Prasad PVV, Boote KJ, Allen LH Jr., Sheehy JE, Thomas JMG. Species, ecotype and cultivar differences in spikelet fertility and harvest index of rice in response to high temperature stress. Field Crops Res. 2006; 95: 398–411.

[19] Matsui T, Omasa K. Rice (*Oryza sativa* L.) cultivars tolerant to high temperature at flowering: anther characteristics. Ann. Bot. 2002;89: 683–687. 12102523 10.1093/aob/mcf112 PMC4233832

[20] Jagadish S, Crauford PQ, Wheeler TR. Phenotyping parents of mapping populations of rice (*Oryza sativa* L.) for heat tolerance during anthesis. Crop Sci. 2008; 48: 1140–1146.

[21] Jagadish S, Muthurajan R, Oane R, Wheeler T, Heuer S, Bennett J, et al Physiological and proteomic approaches to address heat tolerance during anthesis in rice. J. Exp. Bot. 2010;61: 143–156. 10.1093/jxb/erp289 19858118 PMC2791117

[22] Wu KS, Tanksley SD. Abundance, polymorphism and genetic mapping of microsatellites in rice. Mol Gen Genet.1993; 241:225–235. 7901751 10.1007/BF00280220

[23] Panaud O, McCouch SR, Chen X. Development of microsatellite markers and characterization of simple sequence length polymorphism (SSLP) in rice (*Oryzasativa* L.). Mol Gen Genet.1996; 252:597–607. 8914521 10.1007/BF02172406

[24] Xiao J, Li J, Yuan L, McCouch SR, Tanksley SD. Genetic diversity and its relationship to hybrid performance and heterosis in rice as revealed by PCR based markers. Theor Appl Genet. 1996; 92:637–643. 10.1007/BF00226083 24166385

[25] Olufowote JO, Xu Y, Chen X, Park WD, Beachell HM, Goto M, McCouch SR. Comparative evaluation of within cultivar variation of rice (*Oryzasativa* L.) using microsatellite and RFLP markers. Genome.1997; 40:370–378. 9202415 10.1139/g97-050

[26] Thanh ND, Zheng HG, Dong NV, Trinh LN, Ali ML, Nguyen HT. Genetic variation in root morphology and microsatellite DNA loci in upland rice (*Oryzasativa* L.) from Vietnam. Euphytica. 1999;105:43–51.

[27] Pervaiz ZH, Rabbani MA, Khaliq I, Pearce SR, Malik SA. Genetic diversity associated with agronomic traits using microsatellite markers in Pakistani rice landraces. Electron J Biotechnol.2010;13(3):1–14.

[28] Das B, Sengupta S, Parida SK, Roy B, Ghosh M, Prasad M, et al Genetic diversity and population structure of rice landraces from Eastern and North Eastern States of India. BMC Genetics.2013;14: 71 10.1186/147121561471 . 23945062 PMC3765237

[29] Babu BK, Meena V, Agarwal V, Agrawal PK. Population structure and genetic diversity analysis of Indian and exotic rice (*Oryza sativa* L.) accessions using SSR markers. Mol Biol Rep. 2014; 41(7): 4328–39. 10.1007/s110330143304 24584576

[30] Herrera TG, Duque DP, Almeida IP, Núñez GT, Pieters AJ, Martinez CP, Tohme JM. Assessment of genetic diversity in Venezuelan rice cultivars using simple sequence repeats markers. Electron J Biotechnol.2008; 11(5):1–14.

[31] Yu JM, Pressoir G, Briggs WH, Vroh BI, Yamasaki M, Doebley JF, et al A unified mixed model method for association mapping that accounts for multiple levels of relatedness. Nat Genet. 2006;38(2):203–208. 10.1038/ng1702 16380716

[32] Cao L, Zhao J, Zhan X, Li D, He L, Cheng S. Mapping QTLs for heat tolerance and correlation between heat tolerance and photosynthetic rate in rice. Chinese Journal of Rice Science. 2003; 17(3):223–7.

[33] Chen Q, Yu S, Li C, Mou T. Identification of QTLs for heat tolerance at flowering stage in rice. Sci. Agric. Sin. 2008; 41: 315–321.

[34] Zhang T, Yang L, Jiang K, Huang M, Sun Q, Chen W, et al QTL mapping for heat tolerance of the tassel period of rice. Mol. Plant Breed. 2008; 6:867–873.

[35] Zhang G, Chen L, Xiao G, Xiao Y, Chen X, Zhang S. Bulked Segregant Analysis to Detect QTL Related to Heat Tolerance in Rice (*Oryzasativa* L.) Using SSR Markers. Agricultural Sciences in China. 2009; 8(4): 482–487.

[36] Xiao Y, Pan Y, Luo L, Zhang G, Deng H, Dai L, et al Quantitative trait loci associated with seed set under high temperature stress at the flowering stage in rice. Euphytica. 2011; 178: 331–338.

[37] Cardon LR, Bell JI. Association study designs for complex diseases. Nat Rev Genet. 2001;2(2):91–99. 10.1038/35052543 11253062

[38] Flint-Garcia SA, Thornsberry JM, Buckler ES IV. Structure of linkage disequilibrium in plants. Annu Rev Plant Biol.2003; 54(1):357–374. 10.1146/annurev.arplant.54.031902.134907 14502995

[39] Stich B, Melchinger AE, Frisch M, Maurer HP, Heckenberger M, Reif JC (2005) Linkage disequilibrium in European elite maize germplasm investigated with SSRs. Theor Appl Genet 111(4):723–730. 10.1007/s00122-005-2057-x 15997389

[40] Roy JK, Bandopadhyay R, Rustgi S, Balyan HS, Gupta PK. Association analysis of agronomically important traits using SSR, SAMPL and AFLP markers in bread wheat. Curr Sci. 2006; 90(5):683–689.

[41] Remington DL, Thornsberry JM, Matsuoka Y, Wilson LM, Whitt SR, Doebley J, et al Structure of linkage disequilibrium and phenotypic associations in the maize genome. Proc. Natl. Acad. Sci. 2001; 98(20):11479–11484. 10.1073/pnas.201394398 Res. Paper Series 67: 5 11562485 PMC58755

[42] Breseghello F, Sorrells ME. Association mapping of kernel size and milling quality in wheat (*Triticumaestivum* L.) cultivars. Genetics.2006; 172(2):1165–1177. doi: 10.1534/genetics 105.044586 16079235 10.1534/genetics.105.044586 PMC1456215

[43] Tommasini L, Schnurbusch T, Fossati D, Mascher F, Keller B. Association mapping of Stagonosporanodorum blotch resistance in modern European winter wheat varieties. Theor Appl Genet. 2007; 115(5):697–708. doi: 10.1007/ s00122-007-0601-6 17634916 10.1007/s00122-007-0601-6

[44] Kraakman ATW, Martınez F, Mussiraliev B, van Eeuwijk FA, Niks RE. Linkage disequilibrium mapping of morphological, resistance, and other agronomically relevant traits in modern spring barley cultivars. Mol Breeding.2006; 17(1):41–58. 10.1007/s11032-005-1119-8

[45] Hasan M, Friedt W, Pons-Ku¨hnemann J, Freitag NM, Link K, Snowdon RJ. Association of gene-linked SSR markers to seed glucosinolate content in oilseed rape (*Brassicanapus* ssp. *napus*). Theor Appl Genet.2008;116(8):1035–1049. 10.1007/s00122-008-0733-3 18322671

[46] Zhao JJ, Wang XW, Deng B, Lou P, Wu J, Sun RF, et al Genetic relationships within *Brassica rapa* as inferred from AFLP fingerprints. Theor Appl Genet. 2005;110(7):1301–1314. 10.1007/s00122-005-1967-y 15806345

[47] Jun TH, Van K, Kim MY, Lee SH, Walker DR. Association analysis using SSR markers to find QTL for seed protein content in soybean. Euphytica. 2008;162(2):179–191. 10.1007/s10681-007-9491-6

[48] Abdurakhmonov IY, Abdukarimov A. Application of association mapping to understanding the genetic diversity of plant germplasm resources. Int J Plant Genomics. 2008; 574927 10.1155/2008/574927 18551188 PMC2423417

[49] Agrama HA, Eizenga GC, Yan W. Association mapping of yield and its components in rice cultivars. Mol Breeding. 2007; 19(4):341–356. 10.1007/s11032-006-9066-6

[50] Zhao WG, Jong WC, Soon WK, Jeong HL, Kyung HM, Yong JP. Association analysis of physicochemical traits on eating quality in rice (*Oryzasativa* L.). Euphytica. 2013; 191:9–21.

[51] Muthukumar C, Subathra T, Aiswarya, Gayathri V, Babu RC. Comparative genome-wide association studies for plant production traits under drought in diverse rice (*Oryza sativa* L.) lines using SNP and SSR markers. Current Science.2015;109 (1):139–147.

[52] Anandan A, Mahender A, Pradhan SK, Ali J. 2016 Population Structure, Diversity and Trait Association Analysis in Rice (*Oryza sativa* L.) Germplasm for Early Seedling Vigor (ESV) Using Trait Linked SSR Markers. PLOS ONE 11(3): e0152406 10.1371/journal.pone.0152406 27031620 PMC4816567

[53] Pradhan SK, Barik SR, Sahoo J, Pandit E, Pani D, Anandan A.Comparison of Sub1 markers and its combinations for submergence tolerance and analysis of adaptation strategies of rice in rainfed lowland ecology. Comptes Rendus Biology.2015; 10.1016/j.crvi.2015.06.010. 26321658

[54] Anandan A, Pradhan SK, Das SK, Behera L, Sangeetha G. Differential responses of rice genotypes and physiological mechanism under prolonged deepwater flooding. Field Crops Res. 2015; 172: 153–163.

[55] Murray MG and Thompson WF. Rapid isolation of high molecular weight plant DNA. Nucleic Acids Res. 1980 10 10; 8(19): 4321–4325. 7433111 10.1093/nar/8.19.4321 PMC324241

[56] Liao J, Zhang H, Shao X, Zhong P, Huang Y. Identification on Heat Tolerance in Backcross Recombinant Lines and Screening of Backcross Introgression Lines with Heat Tolerance at Milky Stage in Rice. Rice Science. 2011; 18(4):

[57] Ye C, Argayoso M, Redoña E, Sierra S, Laza M, Dilla C, et al Mapping QTL for heat tolerance at flowering stage in rice using SNP markers. Plant Breed. 2012; 131: 33–41.

[58] Ye C, Fatima AT, Argayoso AM, Marcelino AL, Koh H, Redoña ED, Jagadish KSV, Gregorio GB.Identifying and confirming quantitative trait loci associated with heat tolerance at flowering stage in different rice populations.Genetics. 2015; 16:41, 10.1186/s12863-015-0199-7 25895682 PMC4415243

[59] Bui CB, Pham TTH, Bui P, Tran TN, Nguyen VH, Nguyen TP, et al Quantitative Trait Loci Associated with Heat Tolerance in Rice (*Oryzasativa* L.).Plant Breeding and Biotechnology. 2014; 2:14–24.

[60] Jafar A, Fotokian MH, Fabriki-Orang S. Detection of QTLs Influencing Panicle Length, Panicle Grain Number and Panicle Grain Sterility in Rice (*Oryza sativa* L.) J. Crop Sci. Biotech. 2008 (September); 11 (3): 163–170.

[61] Argayoso MA. Redona E.Ye C, Jagadish K.Mapping of heat tolerance quantitative loci (QTL) at flowering stage in rice (Oryzasativa L) Philippine Journal of Crop Science (Philippines).2011:0116–463, Xttp://agris.fao.org /agris search/search.do? record ID=PH2013000629.

[62] Wei H, Liu J, Wang Y, Huang N, Zhang X, Wang L, et al A dominant major locus in chromosome 9 of rice (*Oryzasativa* L.) confers tolerance to 48°C high temperature at seedling stage. Journal of Heredity. 2013:104(2):287–94. 10.1093/jhered/ess103 23258571

[63] Antonio ANA, Paulo HNR, Marcio EF.Mapping of quantitative trait loci for thermosensitive genic male sterility in *indica* rice Pesq. Agropec. Bras., Brasília. 2005; 40(12): 1179–1188.

[64] Lu H, Redus MA, Coburn JR, Rutger JN, McCouch SR, Tai TH. Population structure and breeding patterns of 145 US rice cultivars based on SSR marker analysis. Crop Sci.2005; 45(1):66–76.

[65] Pritchard JK, Stephens M, Donnelly P. Inference of Population Structure Using Multilocus Genotype Data. Genetics. 2000;155: 945–959. 10835412 10.1093/genetics/155.2.945 PMC1461096

[66] Evanno G, Regnaut S, Goudet J. Detecting the number of clusters of individuals using the software STRUCTURE: a simulation study. Mol Ecol. 2005;14(8):2611–2620. doi: 10.1111/ j.1365-294X.2005.02553.x 15969739 10.1111/j.1365-294X.2005.02553.x

[67] Earl DA and Von HBM. STRUCTURE HARVESTER: a website and program for visualizing STRUCTURE output and implementing the Evanno method. Conservation Genetics Resources.2012; 4 (2):359–361. 10.1007/s12686-011-9548-7

[68] Nei M. Genetic distance between populations. Am Nat.1972; 106:283–292.

[69] Perrier X, Jacquemoud-Collet JP. DARwin software.2006; available at http://darwin.cirad.fr/darwin

[70] Peakall R, Smouse PE. GenAlEx 6.5, Genetic analysis in Excel. Population genetic software for teaching and research—an update BIOINFORMATICS. 2012; 28 (19): 2537–2539 10.1093/bioinformatics/bts460 22820204 PMC3463245

[71] Bradbury PJ, Zhang Z, Kroon DE, Casstevens TM, Ramdoss Y, Buckler ES. TASSEL: software for association mapping of complex traits in diverse samples. Bioinformatics. 2007; 23(19):2633–5. 17586829 10.1093/bioinformatics/btm308

[72] Lin HY, Wu YP, Hour AL, Ho SW, Wei FJ, Hsing YI, et al Genetic diversity of rice germplasm used in Taiwan breeding programs. Bot. Studies. 2012; 53: 363–376.

[73] Tenorio FA, Ye C, Redoña E, Sierra S, Laza M, Argayoso MA. Screening rice genetic resources for heat tolerance. SABRAO Journal of Breeding and Genetics.2013; 45 (3):341–51.

[74] Singh N, Choudhury DR, Singh AK, Kumar S, Srinivasan K, Tyagi RK, et al Comparison of SSR and SNP markers in estimation of genetic diversity and population structure of Indian rice varieties. PLOS ONE. 2013; 10.1371/journal.pone.0084136 PMC3868579 24367635

[75] Shah SM, Naveed SA, Arif M. Genetic diversity in basmati and nonbasmati rice varieties based on microsatellite markers. Pak J Bot.2013;45: 423–431.

[76] Chen H, He H, Zou Y, Chen W, Yu R, Liu X, et al Development and application of a set of breeder-friendly SNP markers for genetic analyses and molecular breeding of rice (*Oryzasativa* L.). Theor Appl Genet. 2011; 123(6):869–879. 10.1007/s00122-011-1633-5 21681488

[77] Zhang P, Li J, Li X, Liu X, Zhao X, Lu Y. Population structure and genetic diversity in a rice core collection (*Oryza sativa* L.) investigated with SSR markers. PLOS ONE. 2011 6: e27565 10.1371/journal.pone.0027565 . 22164211 PMC3229487

[78] Agrama HA, Eizenga GC. Molecular diversity and genome-wide linkage disequilibrium patterns in a worldwide collection of *Oryzasativa* and its wild relatives. Euphytica. 2008; 160(3):339–355. 10.1007/s10681-007-9535-y

[79] Jin L, Lu Y, Shao YF, Zhang G, Xiao P, Shen SQ, et al Molecular marker assisted selection for improvement of the eating, cooking and sensory quality of rice (*Oryzasativa* L.). J Cereal Sci. 2010;51:159–164. 10.1016/j.jcs.2009.11.007

[80] Garris AJ, McCouch SR, Kresovich S. Population structure and its effect on haplotype diversity and linkage disequilibrium surrounding the *xa5* locus of rice (*Oryzasativa* L.). Genetics. 2003;165(2):759–769. 14573486 10.1093/genetics/165.2.759 PMC1462795

[81] Salgotra RK, Gupta BB, Bhat JA and Sharma S. Genetic diversity and population structure of basmati rice (*Oryzasativa* L.) Germplasm Collected from North Western Himalayas Using Trait Linked SSR Markers. PLOS ONE.2015; 10.1371/journal.pone.0131858 PMC4517777 26218261

[82] Gebhardt C, Ballvora A, Walkemeier B, Oberhagemann P, Schüler K. Assessing genetic potential in germplasm collections of crop plants by marker-trait association: a case study for potatoes with quantitativevariation of resistance to late blight and maturity type.Molecular Breeding. 2004;13:93–102.

[83] Caicedo AL, Williamson SH, Hernandez RD, Boyko A, Fledel-Alon A, York TL, et al Genome-wide patterns of nucleotide polymorphism in domesticated rice. PLOS Genet. 2007;3(9):e163. 10.1371/journal.pgen.0030163 PMC1994709 17907810

[84] Nachimuthu VV, Muthurajan R, Duraialaguraja S, Sivakami R, Pandian BA, Ponniah G, et al Analysis of Population Structure and Genetic Diversity in Rice Germplasm Using SSR Markers: An Initiative Towards Association Mapping of Agronomic Traits in *Oryzasativa*.Rice. 2015;8:30, 10.1186/s12284-015-0062-5 26407693 PMC4583558

[85] Mather DE, Hyes PM, Chalmers KJ, Eglinton J, Matus I, Richardson K, et al. Use of SSR marker data to study linkage disequilibrium and population structure in Hordeum vulgare: prospects for association mapping in barley. In: International barley genetics symposium. 2004; Brno, Czech Republic, pp. 302–307.

[86] Koyama ML, Levesley A, Koebner RMD, Flowers TJ, Yeo AR. Quantitative trait loci for component physiological traits determining salt tolerance in rice. Plant Physiol. 2001; 25(1):406–422. 10.1104/pp.125.1.406 PMC61021 11154348

