# Supplementary material for: Population Structure, Genetic Diversity and Molecular Marker-Trait Association Analysis for High Temperature Stress Tolerance in Rice
Source: PLoS One. 2016 Aug 5;11(8):e0160027. doi: 10.1371/journal.pone.0160027 (PMC4975506; doi:10.1371/journal.pone.0160027)
Supplement: S1 Fig — (DOCX) [file pone.0160027.s001.docx]

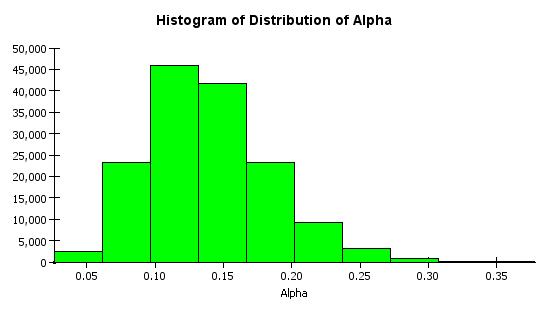


(a)


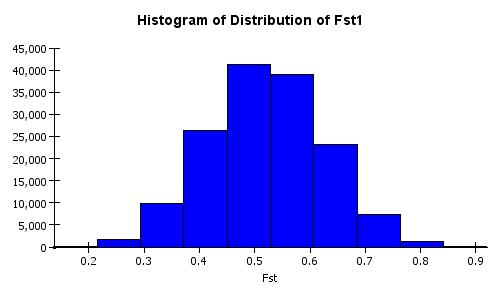


(b)


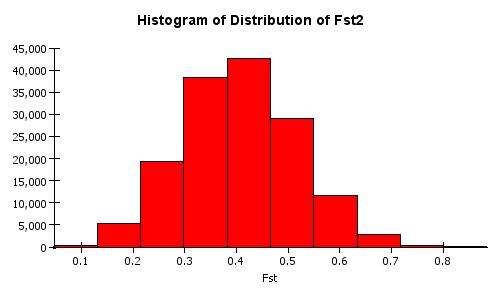


(c)


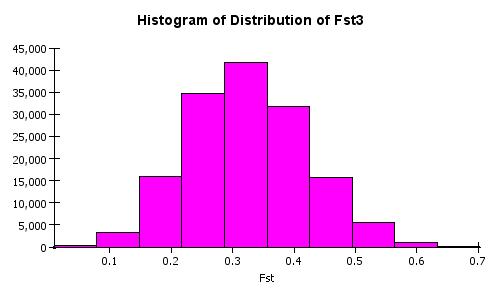


Supplementary fig.1 The distribution pattern of α value in the population and distribution of F_ST_ values in the sub-populations (a)Histogram of distribution of alpha values, (b) Histogram of distribution of Fst1 values, (c) Histogram of distribution of Fst2 values and (d) Histogram of distribution of Fst3 values.
