# Supplementary material for: Population Structure, Genetic Diversity and Molecular Marker-Trait Association Analysis for High Temperature Stress Tolerance in Rice
Source: PLoS One. 2016 Aug 5;11(8):e0160027. doi: 10.1371/journal.pone.0160027 (PMC4975506; doi:10.1371/journal.pone.0160027)
Supplement: S1 Table — (DOCX) [file pone.0160027.s002.docx]

**Supplementary Table2** Germplasm lines used in field screening for high temperature stress tolerance and their spikelet fertility under field screening.

| **Sl. No.** | **genotype name** | **Max. temp(^0^C) on the days to 50% flowering** | **Min. temp (^0^C)on the days to 50% flowering** | **Spikelet**  **fertility%** |
| --- | --- | --- | --- | --- |
| 1 | Ac 39804 | 39.6 | 26.9 | 55.5 |
| 2 | Ac 39800 | 38.4 | 27.4 | 57.2 |
| 3 | Ac 39739 | 37.1 | 28.3 | 57.5 |
| 4 | AC10984 | 38.4 | 27.4 | 75.2 |
| 5 | AC10914 | 37.1 | 26.4 | 76.1 |
| 6 | AC39843 | 40.4 | 27.4 | 73.7 |
| 7 | Ac39737 | 38.4 | 27.4 | 59 |
| 8 | Ac 39955 | 39.6 | 26.8 | 56.4 |
| 9 | Ac 39770 | 38.4 | 27.4 | 56.2 |
| 10 | Ac 39769 | 38.4 | 26.9 | 44.7 |
| 11 | Ac39827 | 38.4 | 26.9 | 39.1 |
| 12 | Ac 39794 | 39 | 24.4 | 48.3 |
| 13 | Ac 39933 | 37.4 | 25 | 49.3 |
| 14 | Ac39928 | 39 | 26.4 | 29.5 |
| 15 | Ac 39823 | 40.4 | 26.8 | 28.6 |
| 16 | Ac39795 | 39.4 | 25.4 | 56.7 |
| 17 | Ac39781 | 40.4 | 26.8 | 55.3 |
| 18 | Ac 39773 | 38.4 | 26.9 | 48.9 |
| 19 | Ac39871 | 39.4 | 25.2 | 48.7 |
| 20 | Ac39776 | 41 | 25.5 | 33.3 |
| 21 | Ac39910 | 40.4 | 27 | 37.8 |
| 22 | Ac39733 | 37.4 | 25 | 55.8 |
| 23 | Ac39749 | 41 | 25.5 | 58.7 |
| 24 | Ac 39792 | 38.4 | 28.2 | 8.5 |
| 25 | Ac 10981 | 38.4 | 26.9 | 19.3 |
| 26 | Ac39755 | 40.4 | 27 | 19.5 |
| 27 | Ac10931 | 39.4 | 25.2 | 58.2 |
| 28 | Ac 39760 | 37.4 | 25 | 46 |
| 29 | Ac10816 | 38.4 | 27.4 | 57.4 |
| 30 | Ac11206 | 40.4 | 27 | 8.8 |
| 31 | Ac39834 | 38.4 | 27.4 | 19.4 |
| 32 | Ac10939 | 40.1 | 25.9 | 57.7 |
| 33 | Ac11209 | 37.9 | 26.8 | 7.6 |
| 34 | Ac 10914 | 38.3 | 26 | 17.9 |
| 35 | AC11261 | 37.7 | 26.6 | 77.6 |
| 36 | Ac 10995 | 35.4 | 26.8 | 48 |
| 37 | Ac10820 | 37 | 25.9 | 57.9 |
| 38 | Ac10837 | 38 | 28.3 | 39.3 |
| 39 | Ac10843 | 39.4. | 25.4 | 38.5 |
| 40 | Ac10840 | 38.4 | 26.9 | 38.8 |
| 41 | Ac11087 | 39.4 | 25.2 | 8.2 |
| 42 | Ac11074 | 41.1 | 28.2 | 19.9 |
| 43 | Ac10990 | 38.4 | 27.4 | 38 |
| 44 | Ac11462 | 42 | 26.8 | 59 |
| 45 | Ac10956 | 38.4 | 26.9 | 37.5 |
| 46 | Ac10965 | 39.4 | 25.2 | 59.4 |
| 47 | Ac10875 | 37.4 | 25 | 39.6 |
| 48 | AC11069 | 38.4 | 27.4 | 68.1 |
| 49 | Ac10811 | 39.4 | 25.2 | 17.5 |
| 50 | Ac10857 | 38.4 | 27.4 | 47.2 |
| 51 | Ac10862 | 39 | 28.2 | 43.2 |
| 52 | Ac39750 | 38.9 | 26 | 17.5 |
| 53 | Ac39957 | 38.4 | 28.2 | 59.8 |
| 54 | Ac39911 | 41 | 25.5 | 34.5 |
| 55 | Ac10915 | 37 | 25.9 | 36.8 |
| 56 | Ac39870 | 39.4 | 25.2 | 37.8 |
| 57 | Ac39840 | 38.4 | 26.9 | 59 |
| 58 | Ac10984 | 40.5 | 28.2 | 46.3 |
| 59 | Ac10954 | 40 | 28.2 | 18.7 |
| 60 | Ac10994 | 41 | 25.5 | 18.2 |
| 61 | Ac10958 | 37 | 25.9 | 58.3 |
| 62 | Ac10925 | 39 | 28.2 | 16.8 |
| 63 | AC10976 | 39 | 26.8 | 65.5 |
| 64 | AC39975 | 38.4 | 26.9 | 64.7 |
| 65 | Ac10957 | 38.8 | 28.2 | 35 |
| 66 | Ac11433 | 38.8 | 28.2 | 57.3 |
| 67 | Ac11085 | 38 | 24.4 | 36.9 |
| 68 | Ac11280 | 38.3 | 28.2 | 57.8 |
| 69 | Ac11217 | 39.4 | 25.2 | 58 |
| 70 | AC10925 | 37.4 | 25 | 65.9 |
| 71 | Ac11328 | 37 | 27.8 | 38.1 |
| 72 | Ac11030 | 37 | 25.9 | 39 |
| 73 | Ac11114 | 41 | 25.5 | 15.5 |
| 74 | Ac 11007 | 39.4 | 25.4 | 46.7 |
| 75 | Ac11205 | 37 | 27.8 | 46.5 |
| 76 | Ac 11258 | 38 | 25.2 | 57.8 |
| 77 | Ac11265 | 37.4 | 25 | 55 |
| 78 | AC39890 | 41 | 25.5 | 79.1 |
| 79 | Ac11333 | 39.4 | 25.4 | 38.9 |
| 80 | Ac11246 | 37.4 | 25 | 37 |
| 81 | Ac39963 | 37 | 25.9 | 58 |
| 82 | Ac11057 | 39.1 | 25.2 | 47.3 |
| 83 | Ac11464 | 37.9 | 28.2 | 48.4 |
| 84 | AC11311 | 37.4 | 25 | 63.6 |
| 85 | Ac39753 | 38.4 | 27.4 | 19.9 |
| 86 | Ac10374 | 37 | 25.9 | 18.6 |
| 87 | Ac39762 | 39.4 | 25.4 | 19.5 |
| 88 | AC39973 | 38.4 | 28.2 | 79.2 |
| 89 | Ac39839 | 38.2 | 25.2 | 48.9 |
| 90 | Ac39797 | 39 | 25.2 | 59 |
| 91 | Ac10978 | 37.4 | 25 | 17.8 |
| 92 | Ac 11152 | 39.4 | 25.4 | 56.6 |
| 93 | Ac10972 | 38.4 | 27.4 | 56.8 |
| 94 | Ac 10899 | 38.4 | 26.9 | 18.7 |
| 95 | Ac39759 | 39.3 | 25.2 | 19.2 |
| 96 | Ac39893 | 38.4 | 26.9 | 56.9 |
| 97 | Ac10972 | 38.9 | 25.2 | 28.5 |
| 98 | Ac 10964 | 38.8 | 28.2 | 55.9 |
| 99 | AC11322 | 39.1 | 25.5 | 62.8 |
| 100 | AC10994 | 40.4 | 26.3 | 63.2 |
| 101 | AC39790 | 43 | 27.4 | 79 |
| 102 | Ac10993 | 41 | 26.2 | 29 |
| 103 | Ac10957 | 40.2 | 26.9 | 58.9 |
| 104 | Ac 11208 | 38.7 | 25.9 | 26.3 |
| 105 | Ac11202 | 39.1 | 26.3 | 28.4 |
| 106 | IR10C-137 | 38.8 | 26.7 | 64.2 |
| 107 | IR10C-167 | 40.1 | 26.1 | 76.4 |
| 108 | IR10C-136 | 40.2 | 25 | 64.9 |
| 109 | IR83142-B-36-B | 38.2 | 25.5 | 61.3 |
| 110 | IR10C-108 | 39.5 | 25.6 | 63.2 |
| 111 | IR10C-161 | 39.5 | 25.3 | 66.1 |
| 112 | HHZ17-Y16-Y3-Y1 | 39.9 | 27.1 | 64.1 |
| 113 | IR10C-110 | 40 | 28 | 61.9 |
| 114 | HHZ8-SAL6-SAL3-SAL1 | 40.2 | 28.4 | 61.2 |
| 115 | IR10C-179 | 40.1 | 26.3 | 62.7 |
| 116 | HHZ11-DT7-SAL1-SAL1 | 39.2 | 29 | 67 |
| 117 | IR10C-103 | 39.9 | 28.9 | 64.4 |
| 118 | HHZ12-Y4-DT1-Y2 | 38.5 | 27.7 | 63.2 |
| 119 | IR10C-126 | 38.2 | 25.2 | 62.1 |
| 120 | IR10G-103 | 39.8 | 25.9 | 66.2 |
| 121 | HHZ17-DT6-Y1-DT1 | 41 | 26.7 | 62.3 |
| 122 | HHZ5-DT20-DT2-DT1 | 41.1 | 25.9 | 65.5 |
| 123 | HHZ12-Y4-DT1-Y3 | 41.5 | 25.8 | 62.1 |
| 124 | HHZ5-SAL10-DT3-Y2 | 39.9 | 25.1 | 63.4 |
| 125 | IR83141-B-32-B | 39.2 | 26.7 | 64.1 |
| 126 | IR64197-3B-15-2 | 39.3 | 25.8 | 64 |
| 127 | HHZ5-SAL14-SAL2-Y2 | 39.9 | 25.4 | 63.7 |
| 128 | HHZ8-SAL6-SAL3-Y1 | 38.2 | 26.8 | 61 |
| 129 | IR10C-157 | 40.2 | 28.1 | 61.2 |
| 130 | Lalat | 40.1 | 27.9 | 57.7 |
| 131 | IR 64 | 41 | 27 | 59 |
| 132 | Satyakrishna | 41.2 | 26 | 16.5 |
| 133 | Sahabhagi | 41 | 26.9 | 66.2 |
| 134 | Satabdi | 39.5 | 25.4 | 66.7 |
| 135 | Annapurna | 39.1 | 27.1 | 59.2 |
| 136 | Surendra | 41.5 | 28 | 48.7 |
| 137 | Birupa | 37.9 | 28.1 | 28.3 |
| 138 | Naveen | 38.9 | 25 | 58.2 |
| 139 | IR 72 | 38.2 | 25.1 | 46.6 |
| 140 | CR2340-2 | 39.1 | 25.4 | 74.9 |
| 141 | CR2340-1 | 37.9 | 25.1 | 62.3 |
| 142 | Vijetha | 38 | 25.9 | 47 |
| 143 | Divya | 44 | 26.1 | 39.5 |
| 144 | Pusa 44 | 37.2 | 26.3 | 38 |
| 145 | ADT 43 | 38.4 | 27 | 59 |
| 146 | CR2463-25 | 39.9 | 28.2 | 58 |
| 147 | Khitish | 38.2 | 27.5 | 28.7 |
| 148 | Shaktiman | 38.2 | 27.3 | 46.4 |
| 149 | Bhoi | 39.2 | 26.4 | 29.1 |
| 150 | Indira | 38.7 | 25.6 | 47.8 |
| 151 | Ratna | 38.8 | 26.7 | 48.2 |
| 152 | Konark | 38.2 | 26.5 | 27.4 |
| 153 | Prasad | 39.1 | 26.1 | 29.5 |
| 154 | BORO 4005 | 40 | 25.4 | 71.1 |
| 155 | CR3813-4-4-4-2-2 | 39.2 | 25.6 | 83.4 |
| 156 | Jajati | 39.9 | 25.6 | 28.1 |
| 157 | Radhi | 39 | 25.7 | 48.8 |
| 158 | Sravani | 38.2 | 25.8 | 38.8 |
| 159 | Sasyasree | 39.4 | 25.5 | 38.9 |
| 160 | Gajapati | 37.8 | 25.8 | 29.4 |
| 161 | Bhavani | 38 | 25.9 | 48.8 |
| 162 | IR 50 | 37 | 25.6 | 39.8 |
| 163 | CR3820-4-5-5-3-1 | 39.3 | 26.2 | 82.3 |
| 164 | Tapaswini | 38.2 | 27.3 | 59 |
| 165 | Ananga | 39 | 26.8 | 40 |
| 166 | Chandan | 37.7 | 26.1 | 73.7 |
| 167 | CR3621-6-1-3-1-2 | 37 | 26 | 68.4 |
| 168 | Satabdi | 40 | 25 | 39.9 |
| 169 | Kshira | 41.2 | 25.1 | 48 |
| 170 | CR 2461-9 | 40.1 | 25.2 | 58.2 |
| 171 | IR 8 | 43.3 | 25.1 | 49 |
| 172 | Gouri | 37.6 | 25.6 | 37.8 |
| 173 | Vikramarya | 37 | 26.1 | 38 |
| 174 | N22 | 38.6 | 26.2 | 88.6 |
| 175 | Dular | 44 | 27 | 78.9 |
| 176 | CO 43 | 37 | 27.1 | 29.9 |
| 177 | Kalyani 2 | 37.4 | 26.6 | 47.7 |
| 178 | Kalakeri | 37.9 | 25.9 | 47.8 |
| 179 | Browngora | 37.4 | 25.8 | 53.3 |
| 180 | CR3825-2-1-2-2-4 | 38.1 | 25 | 74.4 |
| 181 | Sneha | 38.1 | 24.9 | 29 |
| 182 | Lalnakanda | 38.2 | 26.1 | 58.9 |
| 183 | CR 143-2-2 | 38.9 | 25.5 | 75.9 |
| 184 | WAB 56-50 | 37.1 | 27.1 | 26.8 |
| 185 | WITA 10 | 39 | 25.6 | 54.4 |
| 186 | Govinda | 41 | 28.8 | 37.8 |
| 187 | CRDhan 601 | 40.2 | 28 | 72.7 |
| 188 | Rasi | 39.3 | 27.1 | 58.5 |
| 189 | CR3825-2-1-2-2-3 | 39.9 | 26.9 | 75.4 |
| 190 | Jyothi | 38.1 | 27 | 37.5 |
| 191 | Nidhi | 37.7 | 25.5 | 36.4 |
| 192 | CR3820-4-5-3-1-3 | 39.6 | 26.4 | 76.8 |
| 193 | ADT 45 | 40.5 | 26 | 54.4 |
| 194 | Ratnagiri 3 | 41 | 27.2 | 37.9 |
| 195 | Mahamaya | 39.9 | 25 | 38.1 |
| 196 | Kushal | 38.6 | 25.2 | 35.5 |
| 197 | CR3621-6-1-3-1-1 | 39 | 27.1 | 84.5 |
| 198 | CR3820-2-1-5-1-2 | 40.2 | 26 | 85.7 |
| 199 | Bhadra | 39.4 | 26.3 | 49.2 |
| 200 | CR3826-8-3-2-1-1 | 39.9 | 25.8 | 69 |
| 201 | Pant Dhan19 | 40.5 | 26.3 | 58 |
| 202 | Pant Dhan18 | 38.7 | 26.1 | 58.8 |
| 203 | ADT 47 | 38.2 | 27 | 57 |
| 204 | Jawahar | 39.1 | 25.8 | 47.4 |
| 205 | Jaya | 39.7 | 25.3 | 26.9 |
| 206 | CR3622-7-3-2-2 | 39.5 | 25.6 | 68.3 |
| 207 | CR3622-7-3-1-1 | 40 | 26.3 | 67.7 |
| 208 | JGL 1798 | 37.6 | 26.6 | 28.6 |
| 209 | IET 19879 | 39.1 | 26 | 28.2 |
| 210 | IET 19985 | 38.8 | 27 | 44.4 |
| 211 | IET 20538 | 39.3 | 25.1 | 48.4 |
| 212 | IET 20526 | 39.4 | 25.8 | 27 |
| 213 | IET 20532 | 39 | 26 | 58.2 |
| 214 | IET 20525 | 38 | 27 | 57.3 |
| 215 | IET 20529 | 40.1 | 25.6 | 56.6 |
| 216 | IET 20559 | 40.3 | 25 | 34.6 |
| 217 | IET 20533 | 39 | 25.5 | 33.2 |
| 218 | IET 20540 | 39.7 | 25.7 | 55.9 |
| 219 | IET 20534 | 40.2 | 25.8 | 29 |
| 220 | IET 20545 | 38.8 | 28 | 55.8 |
| 221 | IET 20556 | 38.5 | 28.1 | 28.9 |
| 222 | IET 20557 | 40.1 | 27 | 54.2 |
| 223 | IET 20535 | 39.9 | 29.2 | 39 |
| 224 | IET 20561 | 39.1 | 25 | 53.2 |
| 225 | IET 20622 | 38 | 29 | 37 |
| 226 | IET 20601 | 38.9 | 26.6 | 33.8 |
| 227 | IET 20614 | 40 | 25.9 | 38 |
| 228 | IET 19140 | 40.8 | 25.4 | 39.1 |
| 229 | IET 19972 | 42 | 25.6 | 25.9 |
| 230 | Heera | 38.9 | 25.9 | 56.5 |
| 231 | Kalinga3 | 38.7 | 25.2 | 27.4 |
| 232 | Geetanjali | 37 | 25.9 | 51.2 |
| 233 | Kamesh | 39.4 | 28.3 | 35.9 |
| 234 | Parijat | 38.7 | 28 | 54.2 |
| 235 | Naveen | 39.1 | 27.2 | 46.6 |
| 236 | Sadavahar | 39 | 25.6 | 33.2 |
| 237 | Vanaprava | 38.4 | 26.2 | 54.7 |
| 238 | Abhisek | 39.3 | 26 | 48.4 |
| 239 | Anjali | 40 | 27.1 | 49.2 |
| 240 | Phalguni | 39.2 | 27 | 34.4 |
